# Cobalt(III)–Macrocyclic Scaffolds with Anti-Cancer Stem Cell Activity

**DOI:** 10.3390/molecules29122743

**Published:** 2024-06-08

**Authors:** Jiaxin Fang, Philipp Gerschel, Kuldip Singh, Ulf-Peter Apfel, Kogularamanan Suntharalingam

**Affiliations:** 1 School of Chemistry, University of Leicester, Leicester LE1 7RH, UK; jf345@leicester.ac.uk (J.F.); ks42@leicester.ac.uk (K.S.); 2 Inorganic Chemistry I, Ruhr-Universität Bochum, Universitätsstr. 150, 44801 Bochum, Germany; philipp.gerschel@ruhr-uni-bochum.de; 3 Fraunhofer UMSICHT, Osterfelder Str. 3, 46047 Oberhausen, Germany

**Keywords:** metallopharmaceuticals, bioinorganic chemistry, cobalt, cancer stem cells, cyclam, 14-membered macrocycles

## Abstract

Cobalt(III) compounds with tetradentate ligands have been widely employed to deliver cytotoxic and imaging agents into cells. A large body of work has focused on using cobalt(III)–cyclam scaffolds for this purpose. Here, we investigate the cytotoxic properties of cobalt(III) complexes containing 14-membered macrocycles related to cyclam. A breast cancer stem cell (CSC) in vitro model was used to gauge efficacy. Specifically, [Co(1,4,7,11-tetraazacyclotetradecane)Cl_2_]^+^ (**1**) and [Co(1-oxa-4,8,12-triazacyclotetradecane)Cl_2_]^+^ (**2**) were synthesised and characterised, and their breast CSC activity was determined. The cobalt(III) complexes **1** and **2** displayed micromolar potency towards bulk breast cancer cells and breast CSCs grown in monolayers. Notably, **1** and **2** displayed selective potency towards breast CSCs over bulk breast cancer cells (up to 4.5-fold), which was similar to salinomycin (an established breast CSC-selective agent). The cobalt(III) complexes **1** and **2** were also able to inhibit mammosphere formation at low micromolar doses (with respect to size and number). The mammopshere inhibitory effect of **2** was similar to that of salinomycin. Our studies show that cobalt(III) complexes with 1,4,7,11-tetraazacyclotetradecane and 1-oxa-4,8,12-triazacyclotetradecane macrocycles could be useful starting points for the development of new cobalt-based delivery systems that can transport cytotoxic and imaging agents into breast CSCs.

## 1. Introduction

Cobalt is a relatively non-toxic essential trace element metal and is a major component of vitamin B12 and other co-enzymes [1]. Cobalt coordination complexes have been widely investigated for their biological activity over the last 50 years [2,3,4]. Doxovir (CTC-96) is a cobalt(III)–Schiff base complex that has recently passed phase II clinical trials for Herpes labialis (or herpes simplex virus 1) treatment [5,6]. It is the first cobalt-based compound to achieve this feat. Doxovir comprises of cobalt(III) bound to *bis*(acetylacetone)ethylenediimine (an acyclic tetradentate ligand) and two 2-methylimidazole moieties, and is thought to function by forming covalent bonds to key histidine residues within the active site of a viral enzyme that is vital for Herpes replication [7]. Cobalt(III) complexes with cyclic tetradentate ligands have been successfully used as prodrugs and carrier systems to deliver therapeutics and imaging agents into cancer cells, respectively [4,8,9,10]. This approach is reliant on the bioreductive activation of cobalt(III) complexes and the difference in reactivity of the cobalt(III) and cobalt(II) oxidation states [4]. We have used cobalt(III)–cyclam complexes to transport nonsteroidal anti-inflammatory drugs (NSAIDs) into cancer stem cells (CSC) and effect potent, and in some cases, selective CSC death (relative to bulk cancer cells and non-proliferating cells) [11,12,13,14].

CSCs are a sub-population of tumour cells that are pertinent to chemoresistance, metastasis, and relapse [15,16]. Unfortunately, current cancer therapies (chemotherapy, radiation, surgery, and immunotherapy) are unable to completely rid cancer patients of CSCs [17,18]. CSCs surviving treatment are able to generate new tumours in the primary site or in distant organs, due to their self-renewal and differentiation propensity [19]. The academic- and industry-led quest for small molecules and biologics that can remove CSCs at safe doses has yet to identify a suitable clinically appropriate candidate [17]. We and others have developed several metal complexes capable of potently killing CSCs in in vitro and in vivo systems [20,21,22,23]. In particular, the use of cobalt(III)–cyclam prodrugs has yielded fruitful results [11,12,13,14]. Cobalt(III)–cyclam complexes with NSAIDs (naproxen, tolfenamic acid, and flufenamic acid) were shown to kill breast CSCs in the sub-micromolar to nanomolar range [11,12,13,14]. In the oxidised form, the cobalt(III)–cyclam–NSAID platforms are inert owing to the high crystal field stabilisation of the cobalt(III) ion. In the reducing CSC intracellular environment, the cobalt(III) centre undergoes one-electron reduction from cobalt(III) to cobalt(II), resulting in the release of the NSAID moiety, and a cytotoxic cobalt(II) complex capable of damaging genomic DNA via hydrolytic pathways [11,12,13,14]. NSAIDs are established inhibitors of cyclooxygenase-2 (COX-2) [24]. COX-2 catalyses the formation of prostaglandin (an inflammation mediator) and is overexpressed in certain CSCs where it plays a regulatory role [25,26,27]. COX-2 inhibition sensitises CSCs to the cytotoxic reduced cobalt(II) form. We very recently showed that COX-2 inhibition by a cobalt(III)–cyclam complex containing two flufenamic acid units evokes immunogenic cell death of CSCs (through modulation of the inhibitory damage-associated molecular pattern axis) [14]. This was the first cobalt complex of any geometry or oxidation state to display both cytotoxic and immunogenic activating effects on CSCs [14]. Despite our efforts to develop anti-CSC cobalt(III) complexes, we have not investigated the anti-CSC properties of cobalt(III) complexes containing 14-membered macrocycles other than cyclam. Here, we have sought to synthesise, characterise, and evaluate the anti-CSC properties of [Co(isocyclam)Cl_2_]^+^ (**1**) and [Co(oxo-isocyclam)Cl_2_]^+^ (**2**) (where isocylam is 1,4,7,11-tetraazacyclotetradecane and oxo-isocyclam is 1-oxa-4,8,12-triazacyclotetradecane) and compare the data to [Co(cyclam)Cl_2_]^+^ (**3**). The replacement of a nitrogen atom in [Co(isocyclam)Cl_2_]^+^ (**1**) for an oxygen atom to give [Co(oxo-isocyclam)Cl_2_]^+^ (**2**) is expected to better control the reduction of cobalt(III) to cobalt(II). Oxygen is expected to better stabilise the cobalt(III) oxidation state, and thus modulate intracellular bioreduction and overall stability in cell culture media. This is therefore expected to perturb potency towards CSCs.

## 2. Results and Discussion

**Figure 1:**
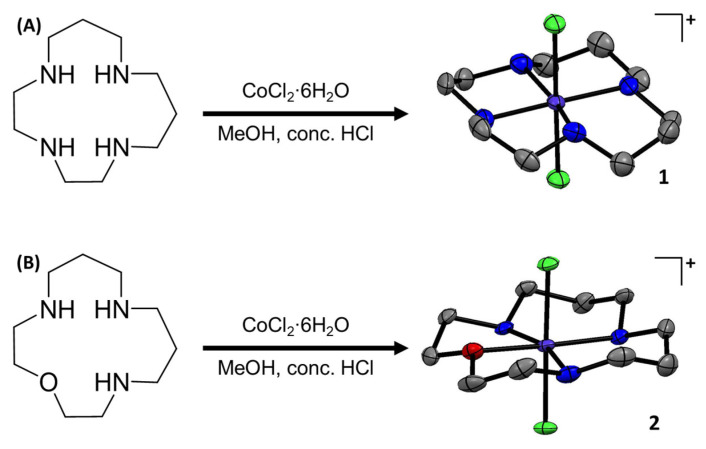
(**A**) Reaction scheme for the preparation of [Co(1,4,7,11-tetraazacyclotetradecane)Cl_2_]Cl (**1**). The X-ray structure of **1** is also shown. Thermal ellipsoids are drawn at 50% probability. C atoms are shown in grey, N in dark blue, Cl in green, and Co in cobalt blue. The H atoms, co-crystallising solvent molecules, and the counter-anion have been omitted for clarity. (**B**) Reaction scheme for the preparation of [Co(1-oxa-4,8,12-triazacyclotetradecane)Cl_2_]½CoCl_4_ (**2**). The X-ray structure of **2** is also shown. Thermal ellipsoids are drawn at 50% probability. C atoms are shown in grey, N in dark blue, Cl in green, O in red, and Co in cobalt blue. The H atoms and the counter-anion have been omitted for clarity.

The chemical structures of the cobalt(III) complexes investigated in this study, [Co(isocyclam)Cl_2_]^+^ (**1**), [Co(oxo-isocyclam)Cl_2_]^+^ (**2**), and [Co(cyclam)Cl_2_]^+^ (**3**), are depicted in Figure 1 and Figure S1. The cobalt(III) complexes **1** and **2** were prepared by reacting equimolar amounts of CoCl_2_∙6H_2_O and 1,4,7,11-tetraazacyclotetradecane [28] or 1-oxa-4,8,12-triazacyclotetradecane [28], respectively, in methanol in the presence of concentrated HCl. The cobalt(III) complexes **1** and **2** were isolated as green solids in reasonable to excellent yields (50–99%) and fully characterised by ^1^H NMR and infra-red spectroscopy, mass spectrometry, elemental analysis, and single-crystal X-ray crystallography (Figures S2–S10, Tables S1–S3). The ^1^H NMR spectra of **1** in methanol-d_4_ and D_2_O at room temperature indicated the presence of multiple conformations in solution (Figures S2 and S3). At low temperature (238 K), the ^1^H NMR spectrum of **1** in methanol-d_4_ displayed a defined set of peaks corresponding to a single conformation (Figure S4). The spectrum is consistent with the *trans*-II conformation observed in the X-ray crystal structure of **1** (Figure 1). The ^1^H NMR spectra of **2** in methanol-d_4_ and D_2_O at room temperature indicated the presence of a single conformation in solution (Figures S5 and S6). The ATR-FTIR spectra for **1** and **2** displayed signals corresponding to N-H and C-H bond stretches associated to the macrocyclic ligands (Figures S7 and S8). As expected, the ATR-FTIR spectra for 1,4,7,11-tetraazacyclotetradecane and 1-oxa-4,8,12-triazacyclotetradecane displayed similar signals to those observed for **1** and **2**, respectively (Figures S11 and S12). The high-resolution ESI mass spectra of **1** and **2** exhibited distinctive molecular ion peaks with the appropriate isotopic pattern expected for the cationic components of **1** (*m*/*z* = 329.0712) and **2** (*m*/*z* = 330.0552) (Figures S9 and S10). This evidences the complexation of 1,4,7,11-tetraazacyclotetradecane and 1-oxa-4,8,12-triazacyclotetradecane to cobalt(III). [Co(cyclam)Cl_2_]^+^ (**3**) was prepared according to a previously reported method and characterised by ^1^H NMR and infra-red spectroscopy and mass spectrometry (Figures S13–S16) [29]. The purity of **1**–**3** was established by elemental analysis (see ESI).

Single crystals of **1** suitable for X-ray diffraction studies were obtained by slow evaporation of a methanolic solution of **1**, and suitable crystals of **2** were obtained by vapour diffusion of diethyl ether into a methanolic solution of **2** (CCDC 2346593- 2346594, Figure 1, Table S1). Selected bond distances and angles are presented in Tables S2 and S3. The cationic component of **1** and **2** consists of a cobalt(III) centre with a distorted octahedral geometry. The cobalt(III) centre in **1** is coordinated to 1,4,7,11-tetraazacyclotetradecane via four nitrogen-donor atoms in *trans*-II fashion and to two additional axial chloride ligands (Figure 1). Similarly, the cobalt(III) centre in **2** is coordinated to 1-oxa-4,8,12-triazacyclotetradecane via three nitrogen-donor atoms and one oxygen-donor atom also in *trans*-II fashion and to two axial chloride ligands (Figure 1). Within the CoN_4_ equatorial plane (for **1**) and the CoN_3_O equatorial plane (for **2**), the average bond angle between nitrogen/oxygen atoms *cis* to one another is 90.1° for **1** and 90.1° for **2**, and the average axial Cl-Co-Cl bond angle is 179.4° for **1** and 178.4° for **2**, consistent with a distorted octahedral geometry. The average Co-N, Co-O, Co-Cl bond lengths are consistent with the values reported for related cobalt(III) complexes [11,14].

To determine the potential for the cobalt(III) complexes **1**–**3** to enter cells, their lipophilicity was experimentally calculated. Specifically, the lipophilicity of **1**–**3** was determined by gauging the extent to which they partitioned between octanol and water, using inductively coupled plasma mass spectrometry (ICP-MS). The experimentally determined LogP values of **1**–**3** varied from −0.38 to −1.46 (Table S4), suggesting that **1**–**3** were hydrophilic enough to dissolve in biological media (required for performing cell-based studies) and at the same time able to enter dividing cells. Time-course UV–Vis spectroscopy and ESI mass spectrometry studies were carried out to assess the stability of **1**–**3** in solution. In DMSO, the d-d bands associated to **1** and **3** (1 mM) remained unchanged over the course of 72 h at 37 °C (Figures S17 and S18), indicative of stability. In DMSO, the absorbance of the d-d bands associated to **2** (1 mM) decreased slightly; however, their corresponding wavelengths remained unaltered over the course of 72 h at 37 °C (Figure S19), indicative of reasonable stability. The ESI mass spectra of **1**–**3** (40 µM) in H_2_O:DMSO (10:1) displayed a molecular ion peak corresponding to **1**–**3** (329 *m*/*z* for **1** and **3**; 330 *m*/*z* for **2**), with the appropriate isotopic pattern throughout the course of 72 h at 37 °C (Figures S20–S22). This suggests that **1**–**3** are able to remain intact in aqueous solutions.

**Figure 2:**
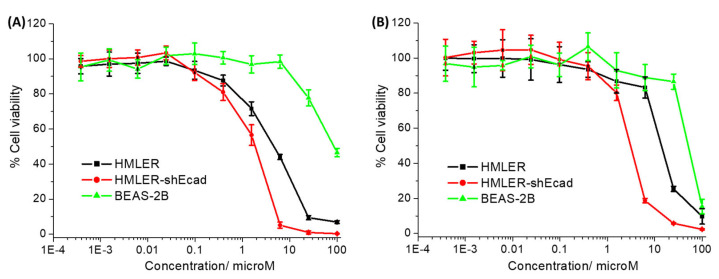
(**A**) Representative dose–response curves for the treatment of HMLER, HMLER-shEcad, and BEAS-2B cells with **1** after 72 h incubation; and (**B**) representative dose–response curves for the treatment of HMLER, HMLER-shEcad, and BEAS-2B cells with **2** after 72 h incubation.

The cytotoxicity of the cobalt(III) complexes **1**–**3** towards bulk breast cancer cells (HMLER) and breast CSCs (HMLER-shEcad) cultured in monolayers was assessed using the colorimetric MTT (3-(4,5-dimethylthiazol-2-yl)-2,5-diphenyltetrazolium bromide) assay. IC_50_ values (the concentration required to reduce cell viability by 50%) were determined from dose–response curves (Figure 2) and are listed in Table 1. The cobalt(III) complexes **1** and **2** displayed micromolar potency towards HMLER and HMLER-shEcad cells. The toxicity of **1** and **2** towards breast CSCs was similar or higher than that of salinomycin (a gold-standard anti-breast CSC agent) and cisplatin (a platinum-based anticancer drug) [30,31]. Based on the IC_50_ values, both **1** and **2** were more toxic towards breast CSCs than bulk breast cancer cells (2.5- and 4.5-fold, respectively). The breast CSC selective potency exhibited by **1** and **2** is similar to that reported for salinomycin [30]. The cobalt(III)–cyclam complex **3** was non-toxic towards HMLER and HMLER-shEcad cells at the concentrations tested (IC_50_ > 100 µM) [11,14]. This implies that modulation of cobalt(III) complexes with 14-membered macrocyclic ligands can result in enhanced potency and selectivity towards breast CSCs. Control cytotoxicity studies indicated that the potency of CoCl_2_, 1,4,7,11-tetraazacyclotetradecane, 1-oxa-4,8,12-triazacyclotetradecane, and cyclam towards HMLER-shEcad cells was significantly lower (*p* < 0.05, *n* = 18) than **1** and **2** (Table S5 and Figure S23). This suggests that the cytotoxicity of **1** and **2** towards breast CSCs is likely to result from the intact cobalt(III) complexes rather than their individual components (cobalt or the free macrocyclic ligand).

To determine the potential of **1** and **2** to kill non-cancerous cells, additional cytotoxicity studies were conducted with epithelial bronchial BEAS-2B cells. The cobalt(III) complexes **1** and **2** were significantly less potent towards BEAS-2B cells than HMLER and HMLER-shEcad cells (IC_50_ value for **1** = 84.18 ± 10.10 μM, up to 46-fold, *p* < 0.05 and IC_50_ value for **2** = 50.58 ± 2.25 μM, up to 16-fold, *p* < 0.05) (Figure 2). Taken together, the cytotoxicity studies in the monolayer systems suggests that **1** and **2** have the potential to preferentially kill breast CSCs and bulk breast cancer cells over non-cancerous bronchial cells.

**Figure 3:**
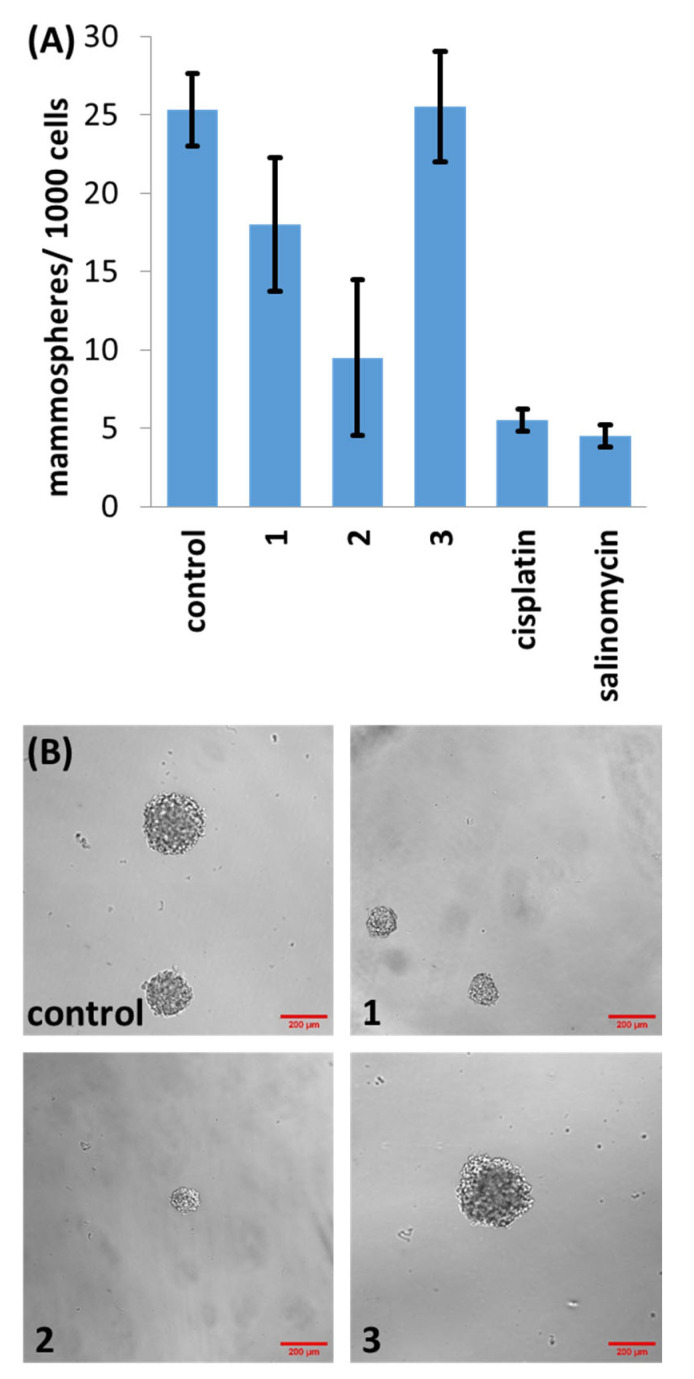
(**A**) Quantification of mammosphere formation with HMLER-shEcad cells untreated and treated with **1**, **2**, **3**, salinomycin or cisplatin (at 2 µM, 5 days). Error bars represent standard deviations; and (**B**) representative bright-field images (×10) of HMLER-shEcad mammospheres in the absence and presence of **1**, **2** or **3** (at 2 µM, 5 days).

Three-dimensional spherical structures akin to tumours called mammospheres can form when breast CSCs are cultured in serum-free, anchorage-independent cell culture conditions [32]. The ability of a given compound to inhibit mammosphere formation and viability is a useful indicator for CSC potency in vivo. The ability of **1**–**3** to inhibit the formation of spherical mammospheres from single suspensions of HMLER-shEcad cells was analysed. Dosages with **1** and **2** (at 2 µM for 5 days) significantly reduced the number and size of the mammospheres formed (Figure 3). Dosage with **3** (at 2 µM for 5 days) did not significantly affect the number or size of mammospheres formed (*p* = 0.45) (Figure 3). Under the same conditions, salinomycin and cisplatin decreased the number of mammospheres formed to a better extent than **1** and **3**, and to a similar extent to **2** (Figure 3). In order to decipher the ability of **1**–**3** to reduce mammosphere viability, the colorimetric resazurin-based reagent TOX8 was used. IC_50_ values (the concentration required to reduce mammosphere viability by 50%) were determined from dose–response curves (Figure S24) and are listed in Table 1. The IC_50_ value of **1** and **2** was in the micromolar range, significantly higher than salinomycin and cisplatin (Table 1) [33,34]. As expected, based on the monolayer studies, **3** was non-toxic towards mammospheres at the concentrations tested (IC_50_ > 133 µM).

**Table 1:** IC_50_ values of the cobalt(III) complexes **1**–**3**, cisplatin and salinomycin against HMLER and HMLER-shEcad cells and HMLER-shEcad mammospheres determined after 72 h or 120 h incubation (mean of two or three independent experiments ± SD).

Compound	HMLER IC_50_ [μM]	HMLER-shEcad IC_50_ [μM]	Mammosphere IC_50_ [μM]
**1**	4.64 ± 0.25	1.83 ± 0.32	51.46 ± 1.49
**2**	13.86 ± 0.01	3.09 ± 0.01	55.04 ± 3.23
**3** ^1^	>100	>100	>133
cisplatin ^1^	2.57 ± 0.02	5.65 ± 0.30	13.50 ± 2.34
salinomycin ^1^	11.43 ± 0.42	4.23 ± 0.35	18.50 ± 1.50

^1^ Taken from references [11,30,31,33,34].

Cell uptake studies were performed to determine the breast CSC permeability of the cobalt(III) complexes **1** and **2**. Specifically, HMLER-shEcad cells were incubated with **1** and **2** (2 µM for 24 h) and the cobalt content was determined by inductively coupled plasma mass spectrometry (ICP-MS). As shown in Figure S25, **1** and **2** were taken up reasonably well by HMLER-shEacd cells, with whole-cell uptake ranging from 51.6 ± 3.2 ng of Co/million cells for **1** to 80.2 ± 4.8 ng of Co/ million cells for **2**. The whole-cell uptake of **1** and **2** is consistent with their relative LogP values (Table S4). The more lipophilic cobalt(III) complex **2** was internalised better by HMLER-shEcad cells than **1**. However, the opposite correlation was noted between whole-cell uptake and cytotoxicity (Table 1). This could be due to the greater redox stability (resistance towards bioactivation) of **2** over **1**, owing to the presence of the oxygen atom which stabilises the cobalt(III) oxidation state.

## 3. Conclusions

In summary we report the synthesis and characterisation of two cobalt(III) complexes with 14-membered macrocyclic ligands (1,4,7,11-tetraazacyclotetradecane for **1** and 1-oxa-4,8,12-triazacyclotetradecane for **2**). The X-ray structures of **1** and **2** show that both adopt a distorted octahedral geometry, with the 14-membered macrocyclic ligands occupying the equatorial positions in a *trans*-II fashion, and the chloride ligands residing in the axial positions. Time course UV–Vis spectroscopy and mass spectrometry studies revealed that **1** and **2** were stable in solution. The cobalt(III) complexes **1** and **2** displayed micromolar potency towards breast CSCs and bulk breast cancer cells, in the same range as salinomycin and cisplatin. Strikingly, **1** and **2** killed breast CSCs selectively compared to bulk breast cancer cells (up to 4.5-fold) and epithelial bronchial cells (up to 46-fold). The cobalt(III) complex **2** was also able to inhibit the formation of mammospheres from a single-cell suspension of breast CSCs to a similar extent as salinomycin. However, the ability of **2** to reduce mammosphere viability was lower than salinomycin. The control cobalt(III)–cyclam complex **3** was non-toxic towards breast CSCs grown in monolayers and in three-dimensional cultures. This suggests that subtle chemical changes to cobalt(III) complexes with 14-membered macrocyclic ligands can facilitate enhanced potency and selectivity towards breast CSCs. Overall, our results show that cobalt(III) complexes with 14-membered macrocyclic ligands can be used to effectively and selectively kill breast CSCs. The results could cultivate further studies that aim to improve the scope of **1** and **2** by using them to deliver cytotoxic and imaging agents into breast CSCs.

## 4. Materials and Methods

### 4.1. General Procedures

All synthetic procedures were performed under normal atmospheric conditions. ^1^H NMR were recorded at room temperature on a Bruker Avance 400 spectrometer (^1^H 400.0 MHz) with chemical shifts (δ, ppm) reported relative to the solvent peaks of the deuterated solvent. Fourier transform infrared (FTIR) spectra were recorded with an IRAffinity-1S Shimadzu spectrophotometer. UV–Vis absorption spectra were recorded on a Cary 3500 UV–Vis spectrophotometer. Inductively coupled plasma mass spectrometry (ICP-MS) was conducted using a Thermo Scientific ICAP-Qc quadrupole ICP mass spectrometer (Waltham, MA, USA). Elemental analysis of the compounds prepared was performed commercially by the University of Cambridge. [Co(cyclam)Cl_2_]Cl, 1,4,7,11-tetraazacyclotetradecane, and 1-oxa-4,8,12-triazacyclotetradecane were prepared using reported protocols [28,29]. CoCl_2_∙6H_2_O and concentrated HCl were purchased from Sigma-Aldrich (St. Louis, MO, USA) and used without further purification. Solvents were purchased from Fisher and used without further purification.

### 4.2. Synthesis of [Co(1,4,7,11-tetraazacyclotetradecane)Cl_2_]Cl (**1**)

A solution of CoCl_2_∙6H_2_O (38.1 mg, 0.16 mmol) in methanol (5 mL) was added to a solution of 1,4,7,11-tetraazacyclotetradecane (32.5 mg, 0.16 mmol) in methanol (10 mL). Concentrated HCl (37%, 1 mL) was added immediately, and the mixture was stirred under reflux conditions for 15 min. The mixture was then stirred at room temperature for 16 h. The resultant solution was filtered with Celite, and diethyl ether (50 mL) was added to the filtrate to produce a precipitate. The precipitate was collected and dried to yield **1** as a light green solid (30 mg, 50%); ^1^H NMR (400 MHz, CD_3_OD) δ_H_ 6.97 (s, 1H), 6.19 (s, 1H), 5.94 (s, 1H), 5.38 (s, 1H), 3.63–3.40 (m, 2H), 3.28–1.81 (m, 18H); ^1^H NMR (400 MHz, D_2_O) δ_H_ 7.04 (s, 1H), 6.22 (s, 1H), 5.96 (s, 1H), 5.44 (s, 1H), 3.48–3.43 (m, 2H), 3.14–1.77 (m, 18H); ATR-FTIR (solid, cm^−1^): 3198, 2961, 2938, 1635, 1462, 1171, 1099, 1083, 1048, 1017, 986, 947, 934, 891, 518, 488, 442, 420, 397; ESI-MS Calcd. for C_10_H_24_Cl_2_CoN_4_ [M-Cl]^+^: 329.0710 a.m.u. Found [M-Cl]^+^: 329.0712 a.m.u.; Anal. Calcd. for C_10_H_24_Cl_3_CoN_4_: C, 32.85; H, 6.62; N, 15.32. Found: C, 32.89; H, 6.70; N, 15.03.

### 4.3. Synthesis of [Co(1-oxa-4,8,12-triazacyclotetradecane)Cl_2_]½CoCl_4_ (**2**)

A solution of CoCl_2_∙6H_2_O (60 mg, 0.25 mmol) in methanol (5 mL) was added to a solution of 1-oxa-4,8,12-triazacyclotetradecane (50 mg, 0.25 mmol) in methanol (10 mL). Concentrated HCl (37%, 1 mL) was added immediately, and the mixture was stirred under reflux conditions for 15 min. The mixture was then stirred at room temperature for 16 h. The resultant solution was filtered with Celite, and diethyl ether (100 mL) was added to the filtrate to produce a precipitate. The precipitate was collected and dried to yield **2** as a dark green solid (107.4 mg, 99%); ^1^H NMR (400 MHz, CD_3_OD) δ_H_ 3.84 (t, 4H), 3.32 (m, 8H), 3.30 (m, 4H), 2.19 (m, 4H); ^1^H NMR (400 MHz, D_2_O) δ_H_ 3.86 (t, 4H), 3.42 (t, 4H), 3.31 (t, 4H), 3.25 (t, 4H), 2.18 (m, 4H); ATR-FTIR (solid, cm^−1^): 3123, 2959, 2798, 2774, 1616, 1593, 1538, 1451, 1433, 1361, 1112, 1056, 1019, 998, 939, 869, 840, 807, 790, 774, 766, 735, 698, 669, 640, 626, 599, 582, 551, 531, 512; ESI-MS Calcd. for C_10_H_23_Cl_2_CoN_3_O [M-½CoCl_4_]^+^: 330.0550 a.m.u. Found [M-½CoCl_4_]^+^: 330.0552 a.m.u.; Anal. Calcd. for C_10_H_23_Cl_4_Co_1.5_N_3_O: C, 27.83; H, 5.37; N, 9.74. Found: C, 28.21; H, 5.99; N, 9.56.

### 4.4. Synthesis of [Co(1,4,8,11-tetraazacyclotetradecane)Cl_2_]Cl (**3**)

[Co(cyclam)Cl_2_]Cl was prepared using a reported protocol [28,29]. Light green solid obtained for **3** (69% yield); ^1^H NMR (400 MHz, CD_3_OD) δ_H_ 6.13 (s, 4H), 2.89–2.80 (m, 12H), 2.53 (dd, 4H), 2.10 (d, 2H), 1.99–1.87 (m, 2H); ^1^H NMR (400 MHz, D_2_O) δ_H_ 6.27 (s, 2H), 3.07–2.47 (m, 18H), 2.26–2.16 (dd, 2H), 1.98–1.88 (d, 2H); ATR-FTIR (solid, cm^−1^): 3161, 2970, 2953, 2937, 2883, 2869, 1472, 1454, 1437, 1423, 1384, 1322, 1295, 1244, 1135, 1102, 1069, 1038, 1013, 904, 890, 813, 556, 523, 505, 445, 422, 402; ESI-MS Calcd. for C_10_H_24_Cl_2_CoN_4_ [M-Cl]^+^: 329.0710 a.m.u. Found [M-Cl]^+^: 329.0717 a.m.u.; Anal. Calcd. for C_10_H_24_Cl_3_CoN_4_: C, 32.85; H, 6.62; N, 15.32. Found: C, 32.96; H, 6.62; N, 15.04.

### 4.5. X-ray Crystallography

Crystals were mounted in inert oil on glass fibres and transferred to a Bruker Apex 2000 CCD area detector diffractometer. Data was collected using graphite-monochromated Mo-Kα radiation (λ = 0.71073) at 150(2) K. Scan type ϖ. Absorption corrections based on multiple scans were applied using SADABS [35] or spherical harmonics implemented in a SCALE3 ABSPACK scaling algorithm [36]. The structures were solved by direct methods and refined on *F*^2^ using the program SHELXT-2016 [37]. All non-hydrogen atoms were refined anisotropically. The CCDC deposition numbers 2346593–2346594 contain the supplementary crystallographic data. This data can be obtained free of charge via The Cambridge Crystallography Data Centre.

### 4.6. Measurement of Water-Octanol Partition Coefficient (LogP)

The LogP value for **1**–**3** was determined using the shake-flask method and inductively coupled plasma mass spectrometry (ICP-MS). The 1-octanol used in this experiment was pre-saturated with water. A DMSO solution of **1**–**3** (10 μL, 10 mM) was incubated with 1-octanol (495 μL) and H_2_O (495 μL) in a 1.5 mL tube. The tube was shaken at room temperature for 24 h. The two phases were separated by centrifugation and the content of **1**–**3** in the water phase was determined by ICP-MS.

### 4.7. Cell Culture

The human mammary epithelial cell lines, HMLER and HMLER-shEcad were kindly donated by Prof. R. A. Weinberg (Whitehead Institute, MIT). HMLER and HMLER-shEcad cells were maintained in Mammary Epithelial Cell Growth Medium (MEGM) with supplements and growth factors (BPE, hydrocortisone, hEGF, insulin, and gentamicin/amphotericin-B). The BEAS-2B bronchial epithelium cell line was acquired from American Type Culture Collection (ATCC, Manassas, VA, USA) and cultured in RPMI 1640 medium with 2 mM L-glutamine supplemented with 1% penicillin and 10% fetal bovine serum. The cells were grown at 310 K in a humidified atmosphere containing 5% CO_2_.

### 4.8. Cytotoxicity Studies: MTT Assay

Exponentially growing cells were seeded at a density of approximately 5 × 10^3^ cells per well in 96-well flat-bottomed microplates and allowed to attach for 24 h prior to the addition of compounds. Various concentrations of the test compounds (0.0004–100 μM) were added and incubated for 72 h at 37 °C (total volume 200 μL). Stock solutions of the compounds were prepared as 10 mM DMSO solutions and diluted using cell media. The final concentration of DMSO in each well was ≤1 %. After 72 h, 20 μL of MTT (4 mg mL^−1^ in PBS) was added to each well and the plates incubated for an additional 4 h at 37 °C. The media/MTT mixture was eliminated and DMSO (100 μL per well) was added to dissolve the formazan precipitates. The optical density was measured at 550 nm using a 96-well multiscanner autoreader. Absorbance values were normalised to (DMSO-containing) control wells and plotted as concentration of compound versus % cell viability. IC_50_ values were interpolated from the resulting dose dependent curves. The reported IC_50_ values are the average of three independent experiments (n = 18).

### 4.9. Tumorsphere Formation and Viability Assay

HMLER-shEcad cells (5 × 10^3^) were plated in ultralow-attachment 96-well plates (Corning, Corning, NY, USA) and incubated in MEGM supplemented with B27 (Invitrogen, Waltham, MA, USA), 20 ng mL^−1^ EGF and 4 μg mL^−1^ heparin (Sigma) for 5 days. Studies were also conducted in the presence of **1**–**3**, cisplatin, and salinomycin (0–133 µM). Mammospheres treated with **1**–**3**, cisplatin, and salinomycin (at 2 µM, 5 days) were counted and imaged using an inverted microscope. The viability of the mammospheres was determined by the addition of a resazurin-based reagent, TOX8 (Sigma). After incubation for 16 h, the fluorescence of the solutions was read at 590 nm (λ_ex_ = 560 nm). Viable mammospheres reduce the amount of the oxidised TOX8 form (blue) and concurrently increase the amount of the fluorescent TOX8 intermediate (red), indicating the degree of mammosphere cytotoxicity caused by the test compound. Fluorescence values were normalised to DMSO-containing controls and plotted as a concentration of the test compound versus % mammosphere viability. IC_50_ values were interpolated from the resulting dose-dependent curves. The reported IC_50_ values are the average of two independent experiments, each consisting of two replicates per concentration level (overall n = 4).

### 4.10. Cellular Uptake

To measure the cellular uptake of **1** and **2**, about 1 million HMLER-shEcad cells were treated with **1** or **2** (2 μM) at 37 °C for 24 h. After incubation, the media were removed and the cells were washed with PBS (2 mL × 3) and harvested. The number of cells was counted at this stage using a haemocytometer. This mitigates any cell death induced by **1** and **2** at the administered concentration and experimental cell loss. The cellular pellet was dissolved in 65% HNO_3_ (250 µL) overnight. Then, the samples were diluted 17-fold with water and analysed using inductively coupled plasma mass spectrometry (ICP-MS, Thermo Scientific ICAP-Qc quadrupole ICP mass spectrometer). Cobalt levels are expressed as mass of Co (ng) per million cells. Results are presented as the mean of two determinations for each data point.

## Data Availability

Samples of the compounds are available from the authors.

## Supplementary Materials

The following supporting information can be downloaded at: https://www.mdpi.com/article/10.3390/molecules29122743/s1, Figures S1–S25; Tables S1–S5.
