# Supplementary material for: Cobalt(III)–Macrocyclic Scaffolds with Anti-Cancer Stem Cell Activity
Source: Molecules. 2024 Jun 8;29(12):2743. doi: 10.3390/molecules29122743 (PMC11206342; doi:10.3390/molecules29122743)
Supplement: Supplementary file 1 [file molecules-29-02743-s001.zip › molecules-3009049-supplementary.pdf]

Supporting Information for

Cobalt(III)–Macrocyclic Scaffolds with Anti-Cancer Stem Cell Activity

Jiaxin Fang [1], Philipp Gerschel [2], Kuldip Singh [1], Ulf-Peter Apfel [2,3,*] and Kogularamanan Suntharalingam [1,*]

1 School of Chemistry, University of Leicester, Leicester LE1 7RH, UK; jf345@leicester.ac.uk (J.F.); ks42@leicester.ac.uk (K.S.)

2 Inorganic Chemistry I, Ruhr-Universität Bochum, Universitätsstr. 150, 44801 Bochum, Germany; philipp.gerschel@ruhr-uni-bochum.de

3 Fraunhofer UMSICHT, Osterfelder Str. 3, 46047 Oberhausen, Germany

* Correspondence: ulf.apfel@rub.de or ulf-peter.apfel@umsicht.fraunhofer.de (U.-P.A.); k.suntharalingam@leicester.ac.uk (K.S.)

Table of Content

Figure S1. Chemical structure of [Co(cyclam)Cl_2]Cl, **3**.
Figure S2. ^{1}H NMR spectrum of **1** in CD_3OD.
Figure S3. ^{1}H NMR spectrum of **1** in D_2O.
Figure S4. Low temperature (238K) ^{1}H NMR spectrum of **1** in CD_3OD.
Figure S5. ^{1}H NMR spectrum of **2** in CD_3OD.
Figure S6. ^{1}H NMR spectrum of **2** in D_2O.
Figure S7. ATR-FTIR spectrum of **1** in the solid form.
Figure S8. ATR-FTIR spectrum of **2** in the solid form.
Figure S9. High resolution ESI mass spectrum (positive mode) of **1**.
Figure S10. High resolution ESI mass spectrum (positive mode) of **2**.
Figure S11. ATR-FTIR spectrum of 1,4,7,11-tetraazacyclotetradecane in the solid form.
Figure S12. ATR-FTIR spectrum of 1-oxa-4,8,12-triazacyclotetradecane in the solid form.
Figure S13. ^{1}H NMR spectrum of **3** in CD_3OD.
Figure S14. ^{1}H NMR spectrum of **3** in D_2O.
Figure S15. ATR-FTIR spectrum of **3** in the solid form.
Figure S16. High resolution ESI mass spectrum (positive mode) of **3**.
Table S1. Crystallographic data for cobalt(III) complexes **1** and **2**.
Table S2. Selected bond lengths (Å) and angles (°) for cobalt(III) complex **1**.
Table S3. Selected bond lengths (Å) and angles (°) for cobalt(III) complex **2**.
Table S4. Experimentally determined LogP values for the cobalt(III) complexes **1-3**.
Figure S17. UV-Vis spectrum of **1** (1 mM) in DMSO over the course of 72 h at 37 ^{o}C.
Figure S18. UV-Vis spectrum of **3** (1 mM) in DMSO over the course of 72 h at 37 ^{o}C.
Figure S19. UV-Vis spectrum of **2** (1 mM) in DMSO over the course of 72 h at 37 ^{o}C.
Figure S20. ESI mass spectra (positive mode) of **1** (40 μM) in H_2O:DMSO (10:1) (A) before and after incubation for (B) 24 h, (C) 48 h, and (D) 72 h at 37 ^{o}C.
Figure S21. ESI mass spectra (positive mode) of **2** (40 μM) in H_2O:DMSO (10:1) (A) before and after incubation for (B) 24 h, (C) 48 h, and (D) 72 h at 37 ^{o}C.
Figure S22. ESI mass spectra (positive mode) of **3** (40 μM) in H_2O:DMSO (10:1) (A) before and after incubation for (B) 24 h, (C) 48 h, and (D) 72 h at 37 ^{o}C.
Figure S23. Representative dose-response curves for the treatment of HMLER-shEcad cells with $CoCl_2$, 1,4,7,11-tetraazacyclotetradecane, 1-oxa-4,8,12-triazacyclotetradecane, or cyclam after 72 h incubation.
Table S5. IC_{50} values of $CoCl_2$, 1,4,7,11-tetraazacyclotetradecane, 1-oxa-4,8,12-triazacyclotetradecane, and cyclam against HMLER-shEcad cells. Determined after 72 h incubation (mean of three independent experiments ± SD).
Figure S24. Representative dose-response curves for the treatment of HMLER-shEcad mammospheres with **1-3** after 5 days incubation.
Figure S25. Cobalt content in HMLER-shEcad cells treated with **1** or **2** (2 μM for 24 h).

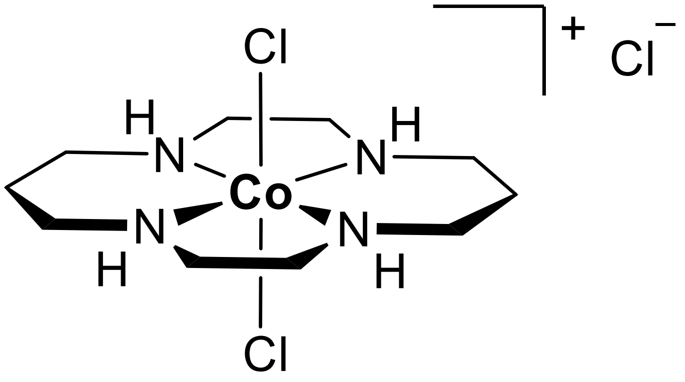

Figure S1. Chemical structure of $[Co(cyclam)Cl_2]Cl$, **3**.

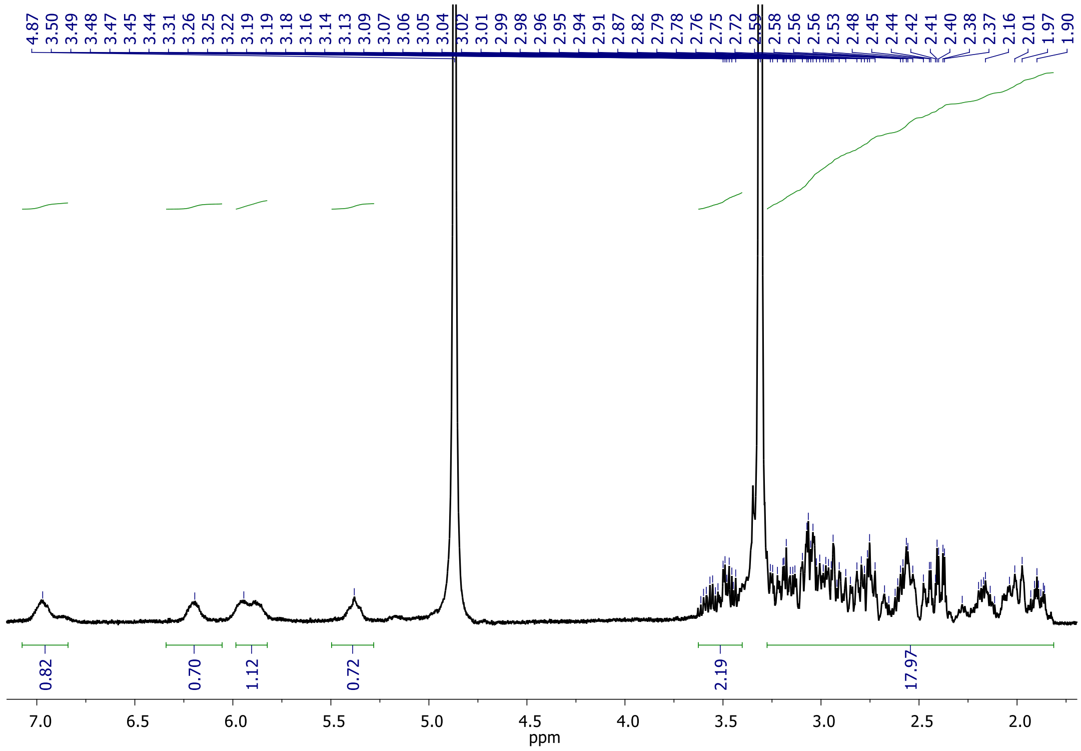

Figure S2. ^{1}H NMR spectrum of **1** in CD_3OD.

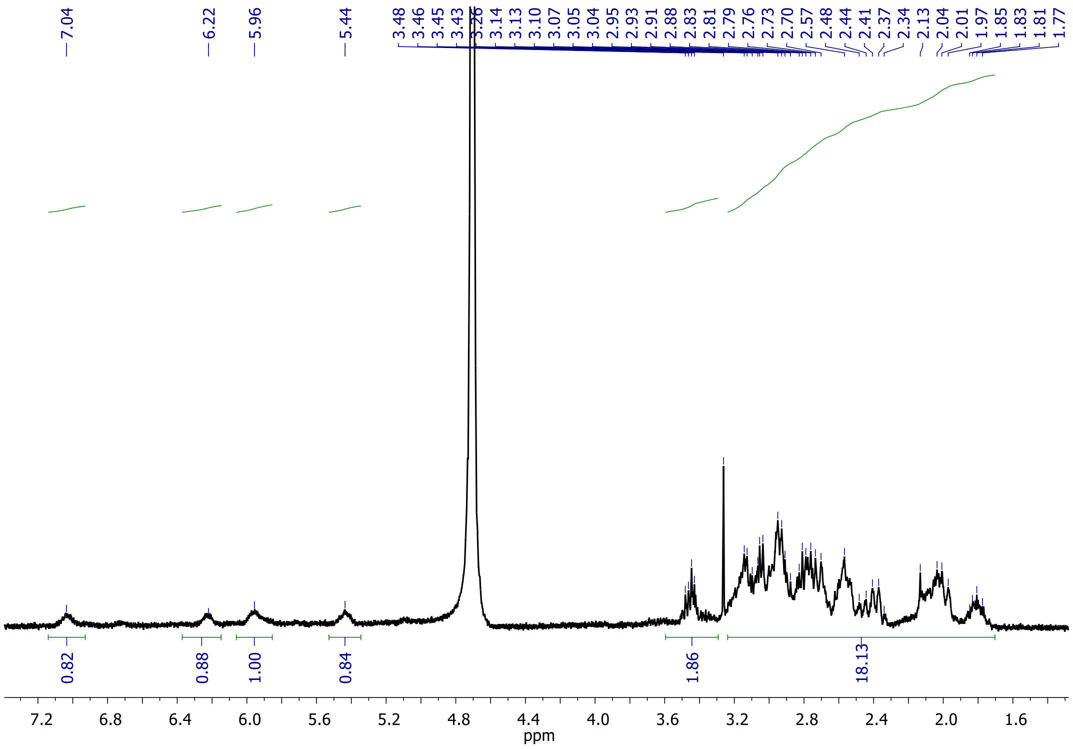

Figure S3. ^{1}H NMR spectrum of **1** in D_2O.

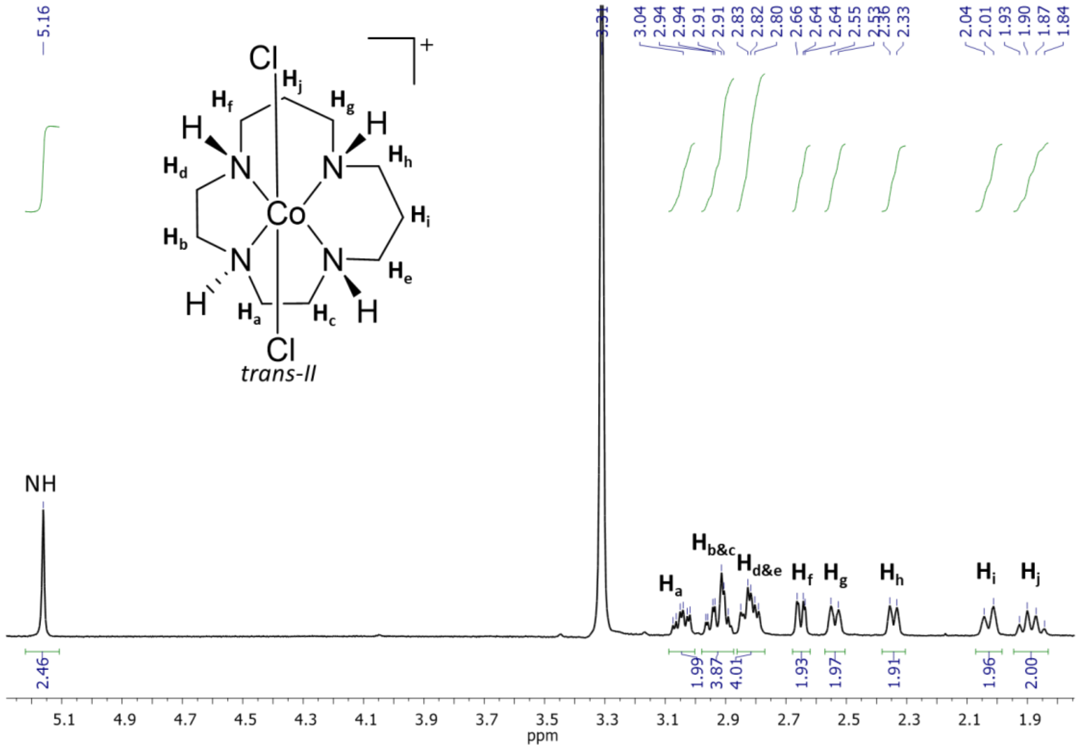

Figure S4. Low temperature (238K) ^{1}H NMR spectrum of **1** in CD_3OD (with tentative NMR assignments).

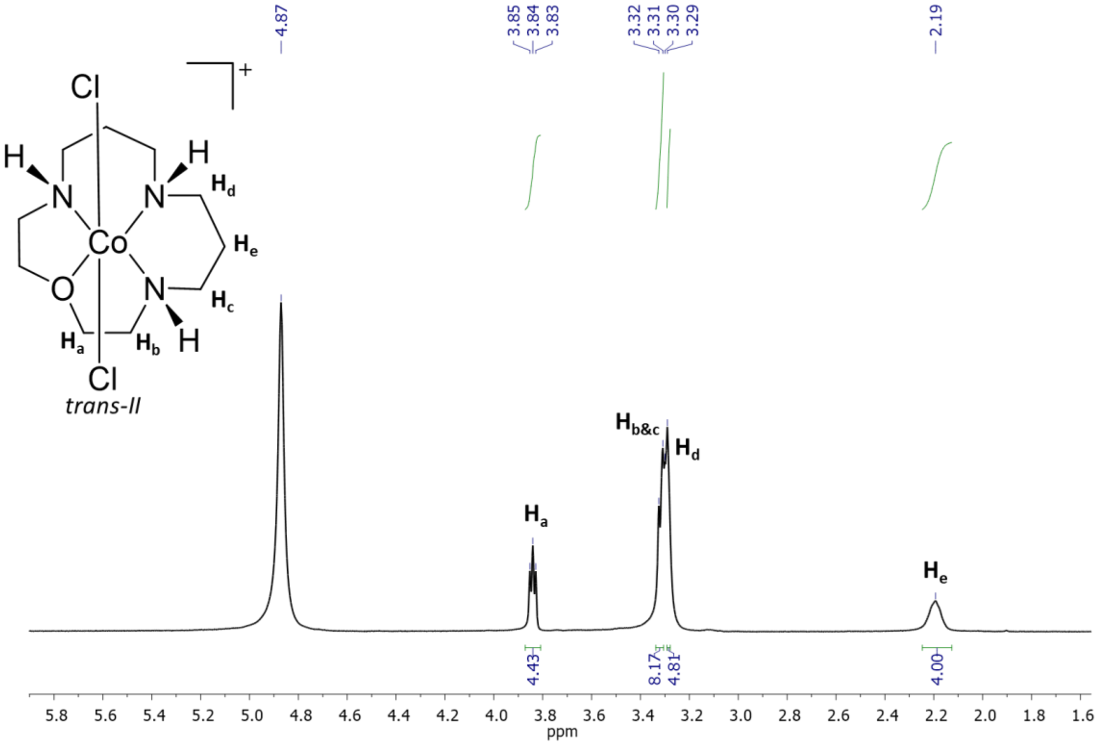

Figure S5. ^{1}H NMR spectrum of **2** in CD_3OD (with tentative NMR assignments).

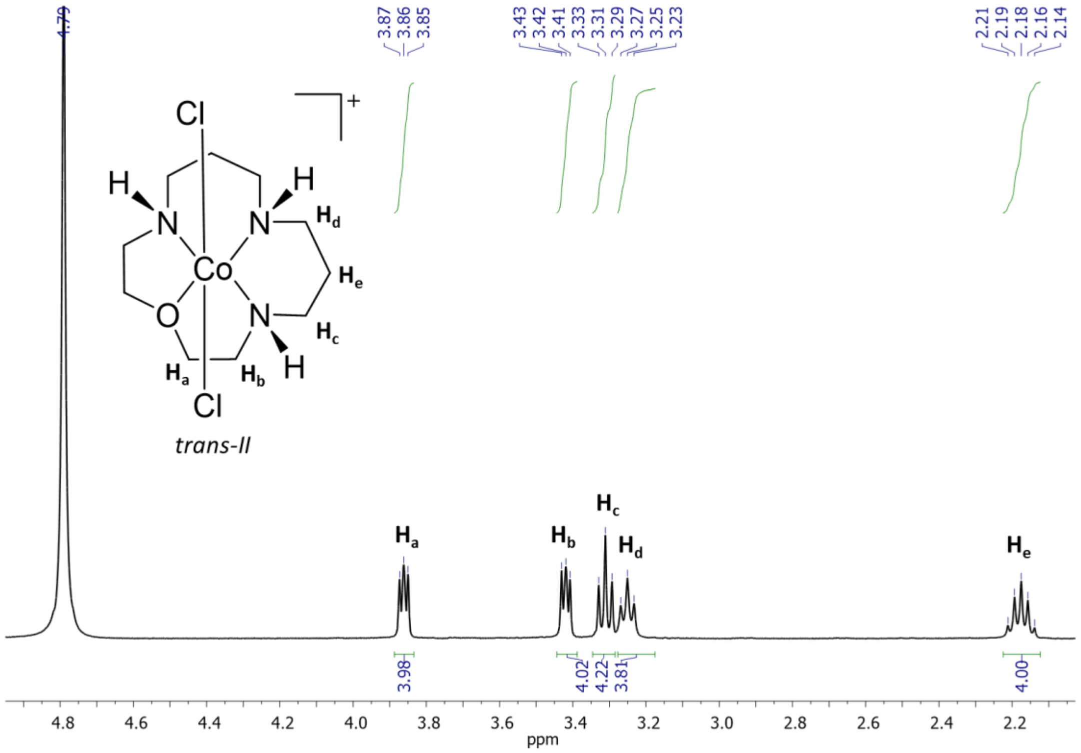

Figure S6. ^{1}H NMR spectrum of **2** in D_2O (with tentative NMR assignments).

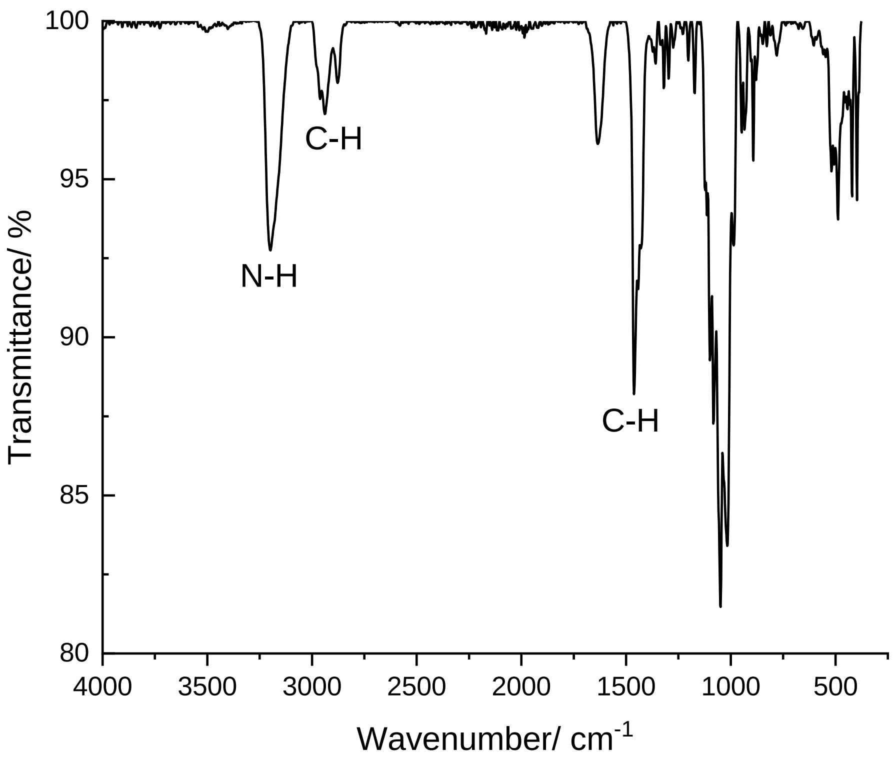

Figure S7. ATR-FTIR spectrum of **1** in the solid form.

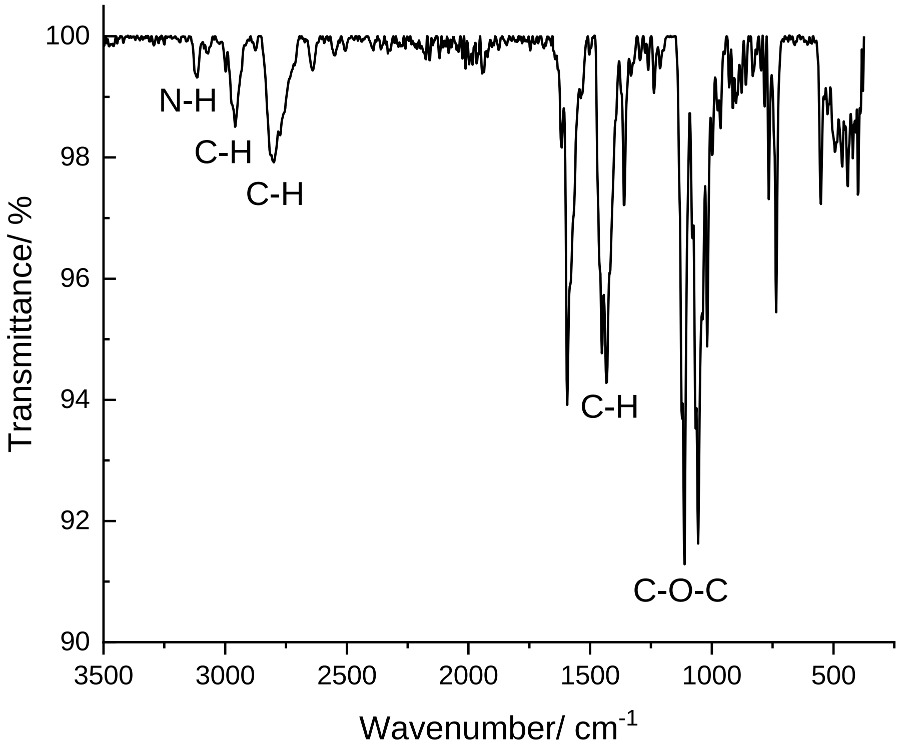

Figure S8. ATR-FTIR spectrum of **2** in the solid form.

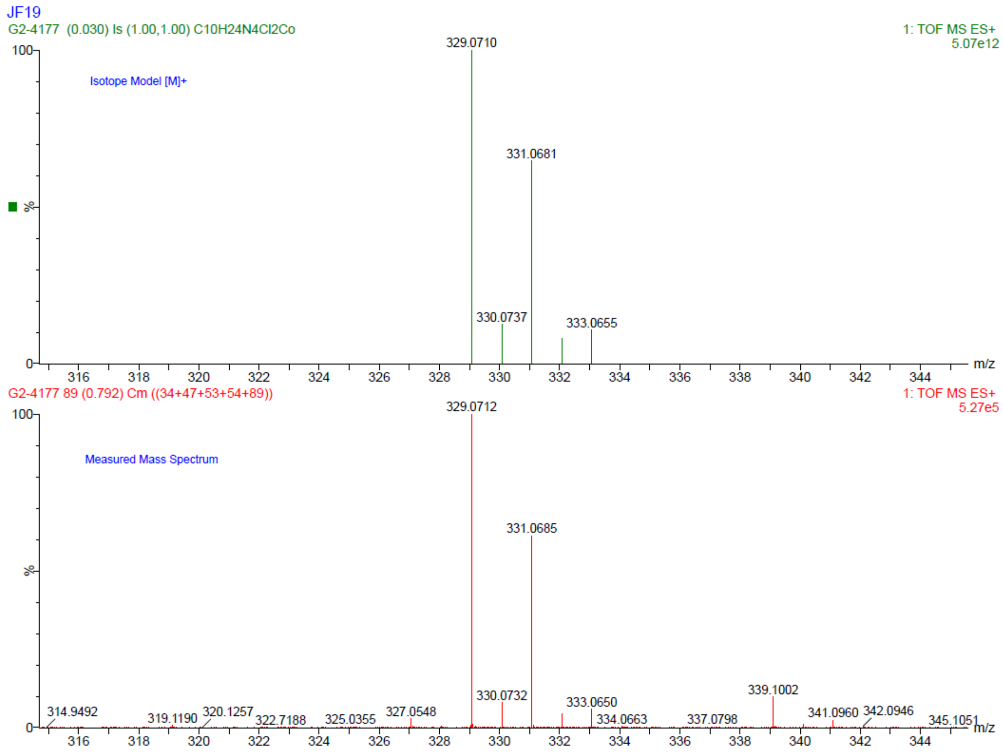

Figure S9. High resolution ESI mass spectrum (positive mode) of **1**.

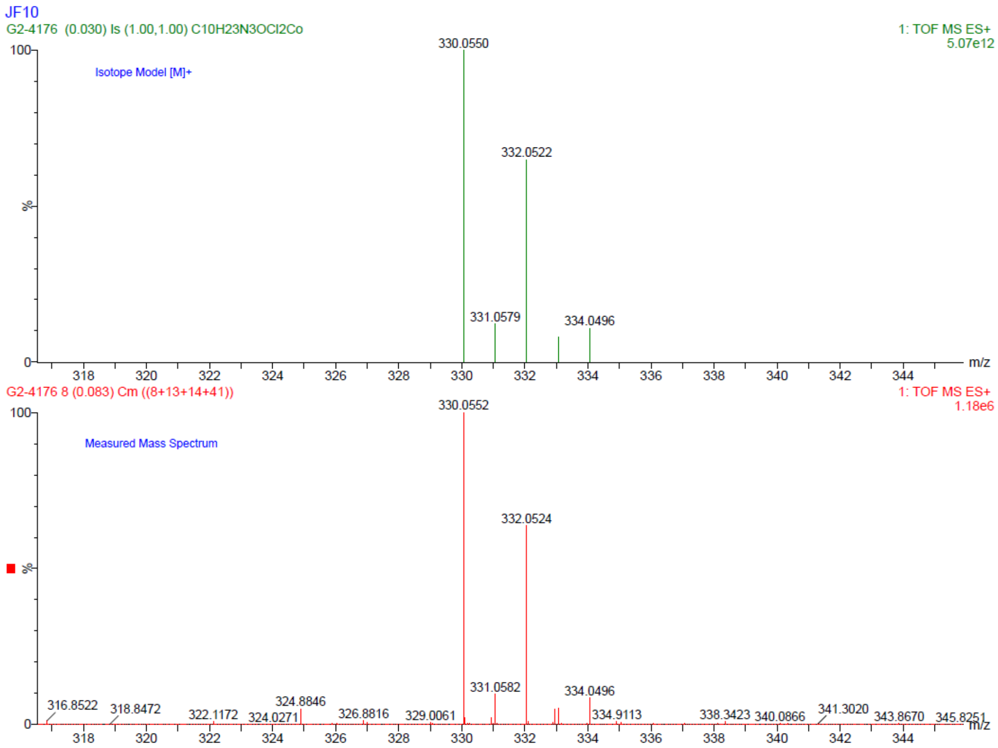

Figure S10. High resolution ESI mass spectrum (positive mode) of **2**.

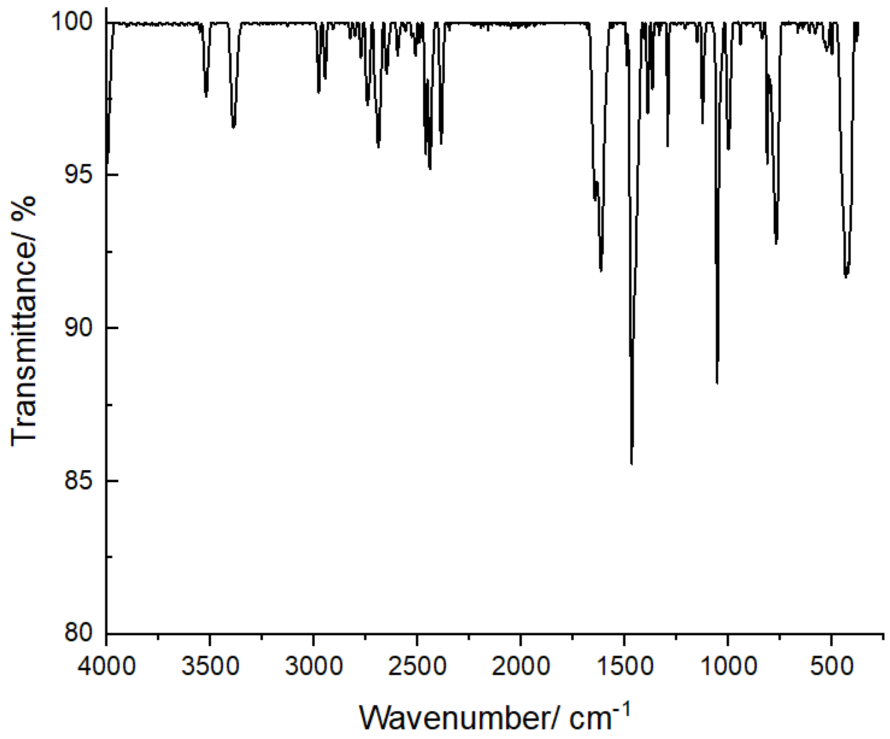

Figure S11. ATR-FTIR spectrum of 1,4,7,11-tetraazacyclotetradecane in the solid form.

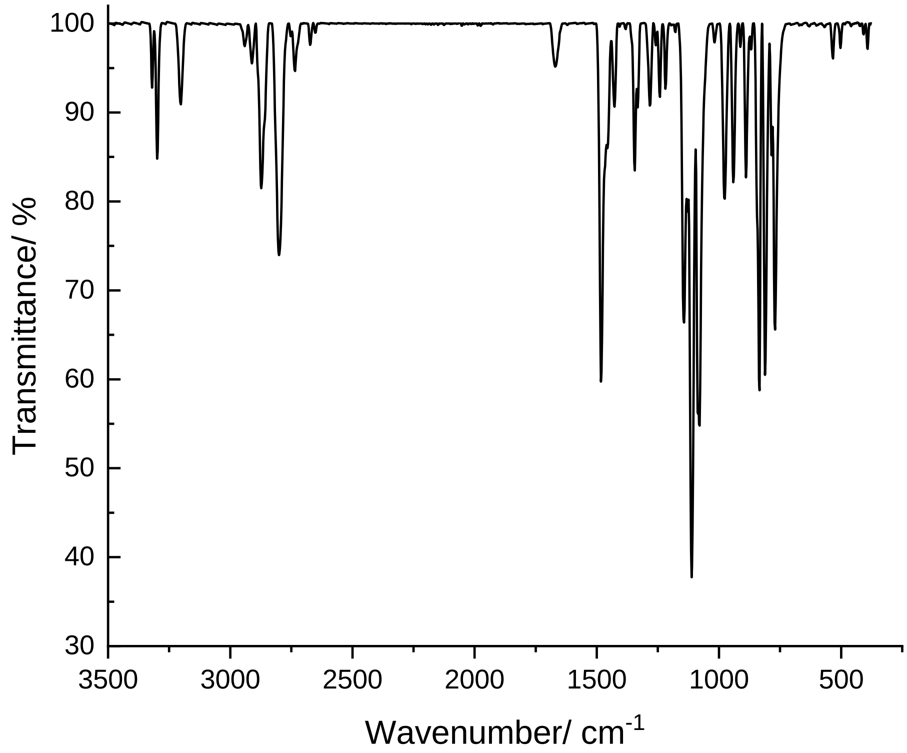

Figure S12. ATR-FTIR spectrum of 1-oxa-4,8,12-triazacyclotetradecane in the solid form.

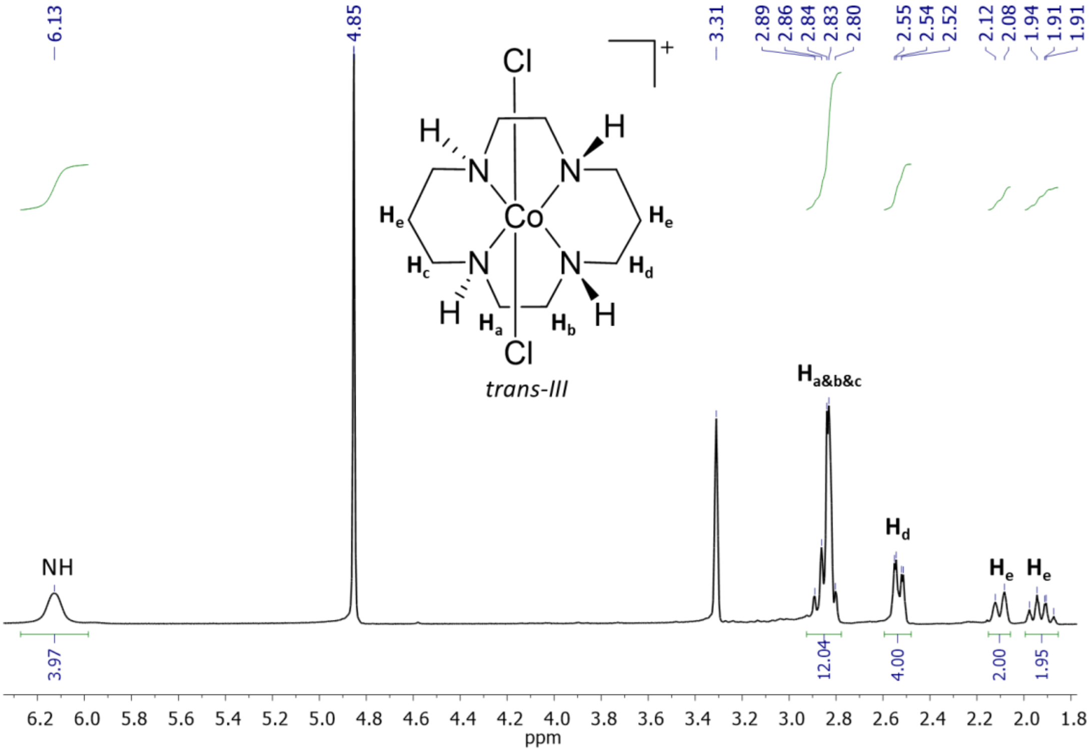

Figure S13. ^{1}H NMR spectrum of **3** in CD_3OD (with tentative NMR assignments).

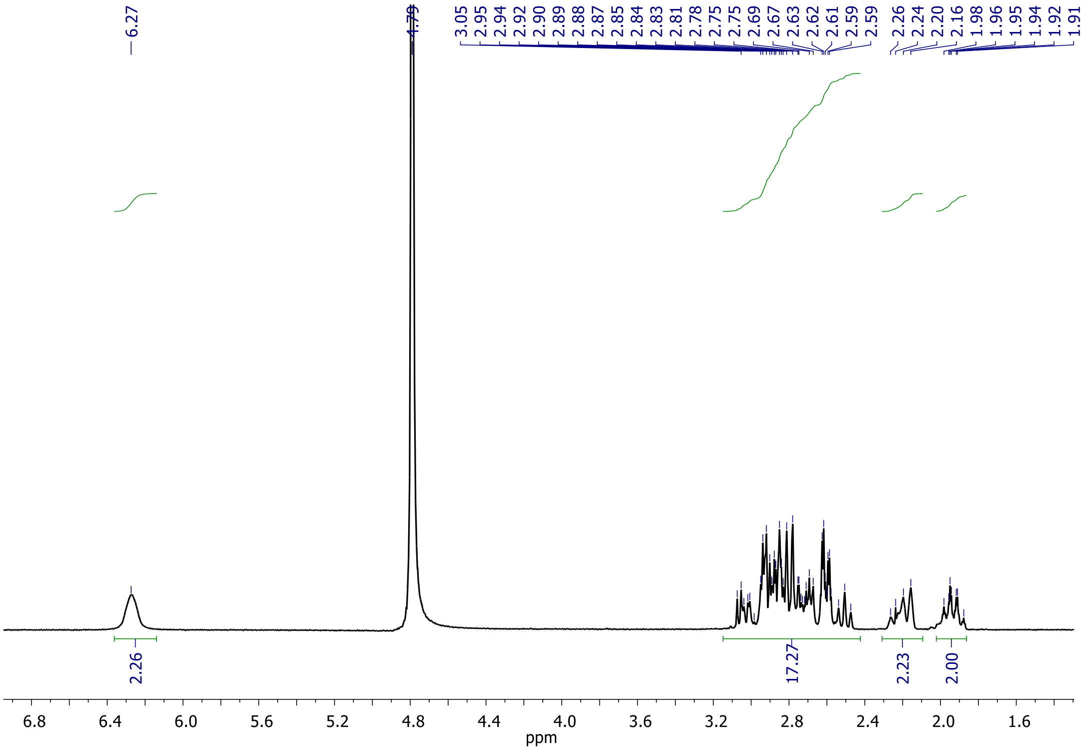

Figure S14. ^{1}H NMR spectrum of **3** in D_2O.

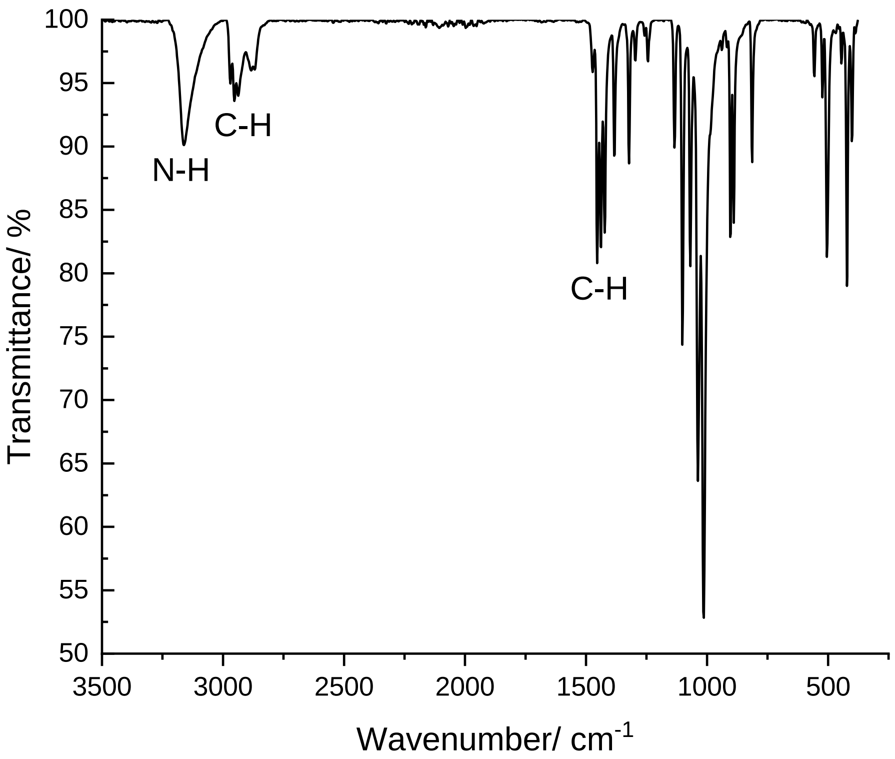

Figure S15. ATR-FTIR spectrum of **3** in the solid form.

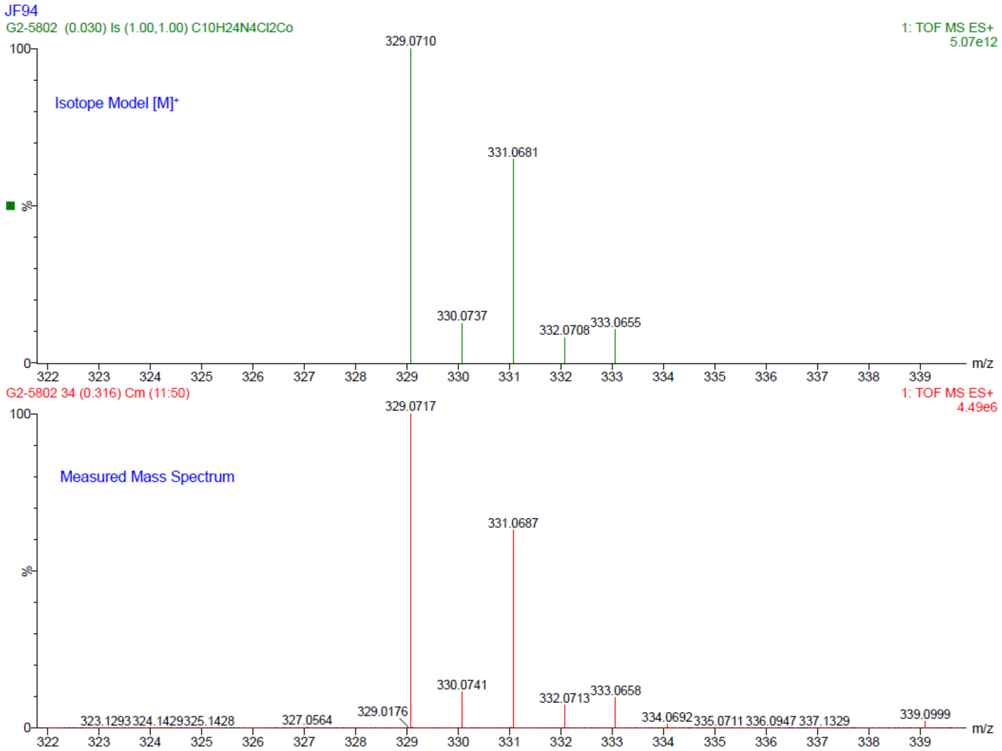

Figure S16. High resolution ESI mass spectrum (positive mode) of **3**.

Table S1. Crystallographic data for cobalt(III) complexes **1** and **2**.

**Metal complex**	**1**	**2**
CCDC No.	2346593	2346594
formula	$C_{10}H_{24}Cl_3CoN_4{\cdot}3H_2O$	$[(C_{10}H_{23}Cl_4CoN_3O)_2]CoCl_4$
Fw	419.66	863.02
Crystal system	orthorhombic	monoclinic
Space group	$Pmn2_1$	$P2_1/c$
a, Å	20.9410(10)	18.0688(6)
b, Å	6.4187(4)	40.2468(13)
c, Å	7.3240(4)	18.8332(6)
α, deg.	90	90
β, deg.	90	91.917(2)
γ, deg.	90	90
V, Å^3	984.45(9)	13688.1(8)
Z	2	16
D_{calcd}, Mg/m^3	1.416	1.675
2θ / deg.	8.444 to 145.446	4.39 to 170.188
Reflections collected	5192	135269
Independent reflections	1571	27157
Goodness-of-fit on F^2	1.097	1.095
R_1, wR_2 [$I \geq 2\sigma(I)$]	0.0677, 0.1939	0.0839, 0.1718
R_1, wR_2 [all data]	0.0701, 0.1969	0.1500, 0.2095

Table S2. Selected bond lengths (Å) and angles (°) for cobalt(III) complex **1**.

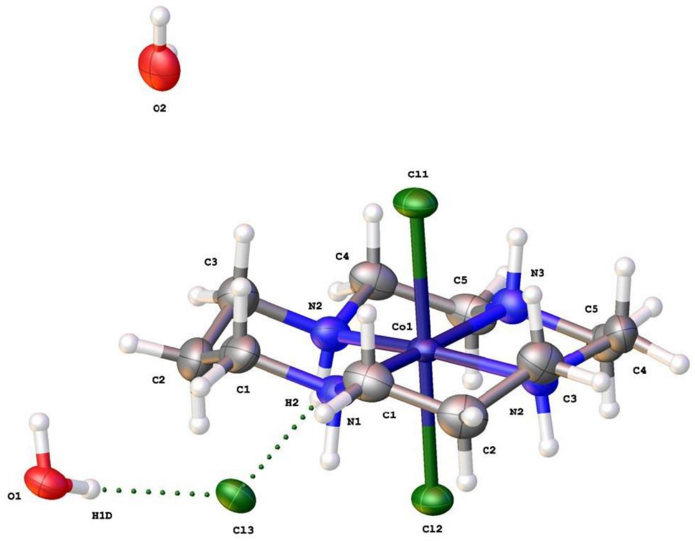

Co(1)-Cl(1)	2.232(4)	Co(1)-N(2)^{1}	1.985(7)
Co(1)-Cl(2)	2.279(4)	Co(1)-N(2)	1.985(7)
Co(1)-N(1)	1.986(11)	Co(1)-N(3)	1.963(12)
Cl(1)-Co(1)-Cl(2)	179.4(2)	N(2)-Co(1)-N(1)	96.2(2)
N(1)-Co(1)-Cl(1)	91.6(4)	N(2)^{1}-Co(1)-N(2)	164.0(5)
N(1)-Co(1)-Cl(2)	87.8(4)	N(3)-Co(1)-Cl(1)	87.4(4)
N(2)^{1}-Co(1)-Cl(1)	94.9(2)	N(3)-Co(1)-Cl(2)	93.2(4)
N(2)-Co(1)-Cl(1)	94.9(2)	N(3)-Co(1)-N(1)	179.0(6)
N(2)^{1}-Co(1)-Cl(2)	85.2(2)	N(3)-Co(1)-N(2)^{1}	83.9(2)
N(2)-Co(1)-Cl(2)	85.2(2)	N(3)-Co(1)-N(2)	83.9(2)
N(2)^{1}-Co(1)-N(1)	96.2(2)		

Table S3. Selected bond lengths (Å) and angles (°) for cobalt(III) complex **2**.

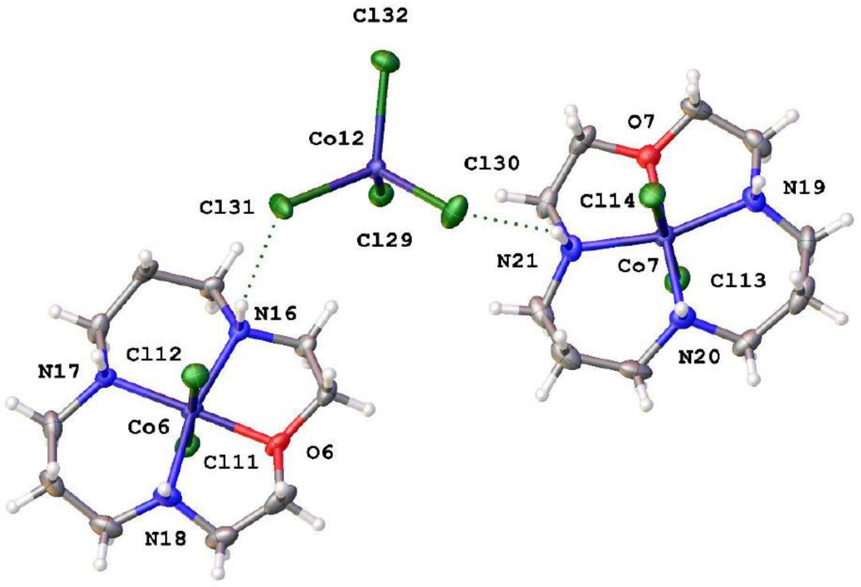

Co(6)-Cl(11)	2.152(2)	Co(7)-Cl(13)	2.157(2)
Co(6)Cl(12)	2.152(2)	Co(7)-Cl(14)	2.169(2)
Co(6)-O(6)	1.967(6)	Co(7)-O(7)	1.973(6)
Co(6)-N(16)	2.053(7)	Co(7)-N(19)	2.066(7)
Co(6)-N(17)	1.990(7)	Co(7)-N(20)	1.988(7)
Co(6)-N(18)	2.049(7)	Co(7)-N(21)	2.026(7)
Cl(11)-Co(6)-Cl(12)	178.82(12)	Cl(13)-Co(7)-Cl(14)	178.07(12)
O(6)-Co(6)-Cl(11)	88.59(19)	O(7)-Co(7)-Cl(13)	88.64(19)
O(6)-Co(6)-Cl(12)	92.48(19)	O(7)-Co(7)-Cl(14)	93.21(19)
O(6)-Co(6)-N(16)	81.5(3)	O(7)-Co(7)-N(19)	81.0(3)
O(6)-Co(6)-N(17)	177.8(3)	O(7)-Co(7)-N(20)	177.9(3)
O(6)-Co(6)-N(18)	84.5(3)	O(7)-Co(7)-N(21)	85.4(3)
N(16)-Co(6)-Cl(11)	94.5(2)	N(19)-Co(7)-Cl(13)	94.2(2)
N(16)-Co(6)-Cl(12)	86.1(2)	N(19)-Co(7)-Cl(14)	86.6(2)
N(17)-Co(6)-Cl(11)	89.3(2)	N(20)-Co(7)-Cl(13)	89.3(2)

N(17)-Co(6)-Cl(12)	89.6(2)	N(20)-Co(7)-Cl(14)	88.8(2)
N(17)-Co(6)-N(16)	98.1(3)	N(20)-Co(7)-N(19)	98.7(3)
N(17)-Co(6)-N(18)	96.2(3)	N(20)-Co(7)-N(21)	95.1(3)
N(18)-Co(6)-Cl(11)	92.2(2)	N(21)-Co(7)-Cl(13)	92.3(2)
N(18)-Co(6)-Cl(12)	87.4(2)	N(21)-Co(7)-Cl(14)	87.3(2)
N(18)-Co(6)-N(16)	164.3(3)	N(21)-Co(7)-N(19)	164.8(3)

Table S4. Experimentally determined LogP values for the cobalt(III) complexes **1-3**.

Cobalt(III) complex	LogP value
**1**	-1.11
**2**	-0.38
**3**	-1.46

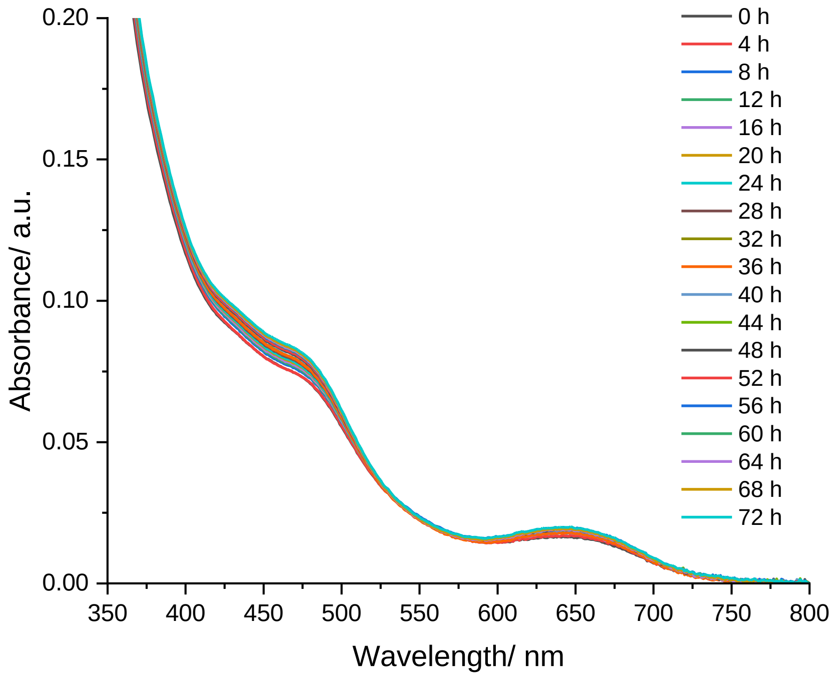

Figure S17. UV-Vis spectrum of **1** (1 mM) in DMSO over the course of 72 h at 37 °C.

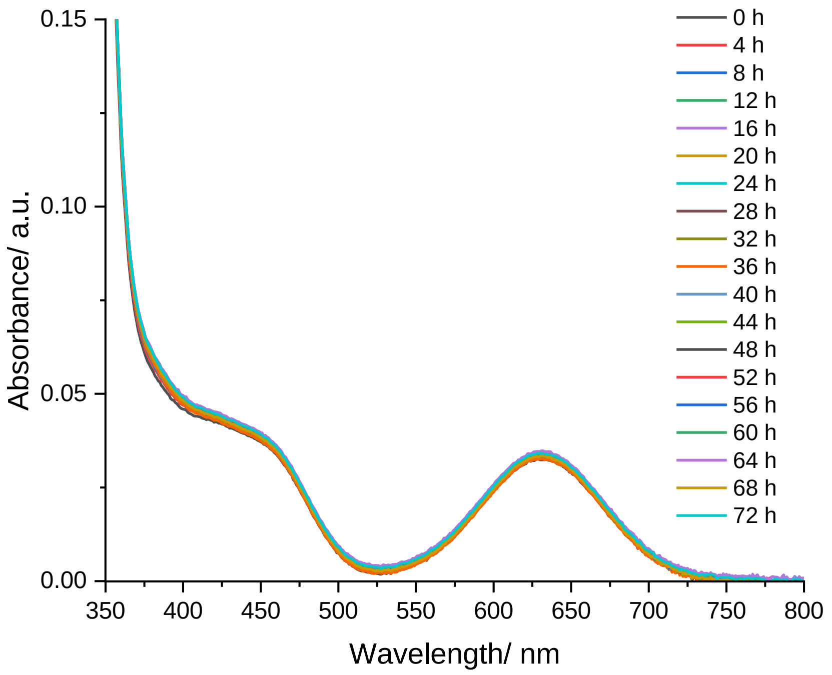

Figure S18. UV-Vis spectrum of **3** (1 mM) in DMSO over the course of 72 h at 37 °C.

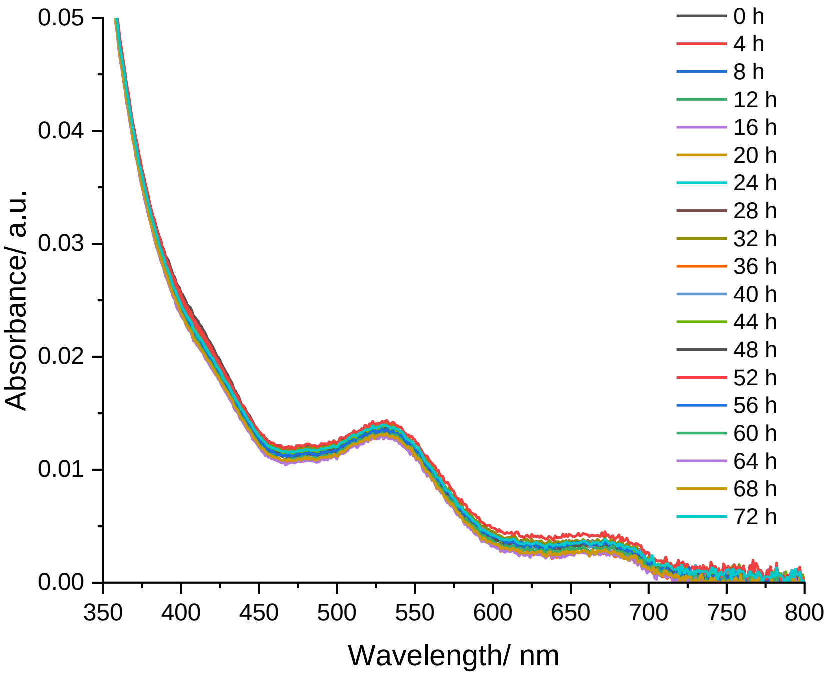

Figure S19. UV-Vis spectrum of **2** (1 mM) in DMSO over the course of 72 h at 37 ^{o}C.

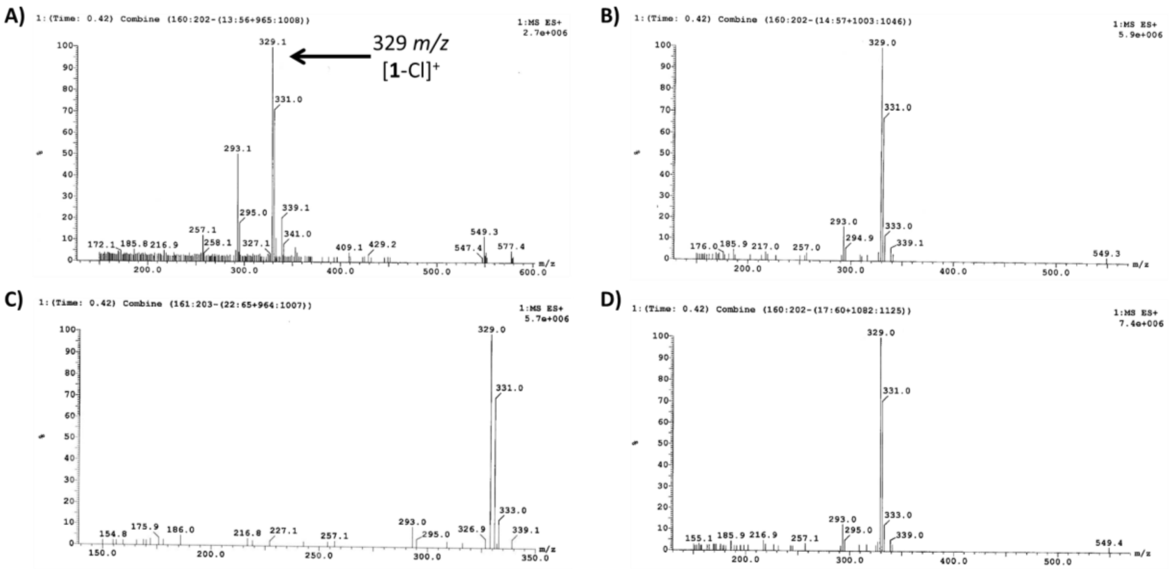

Figure S20. ESI mass spectra (positive mode) of **1** (40 μM) in H_2O:DMSO (10:1) (A) before and after incubation for (B) 24 h, (C) 48 h, and (D) 72 h at 37 ^{o}C.

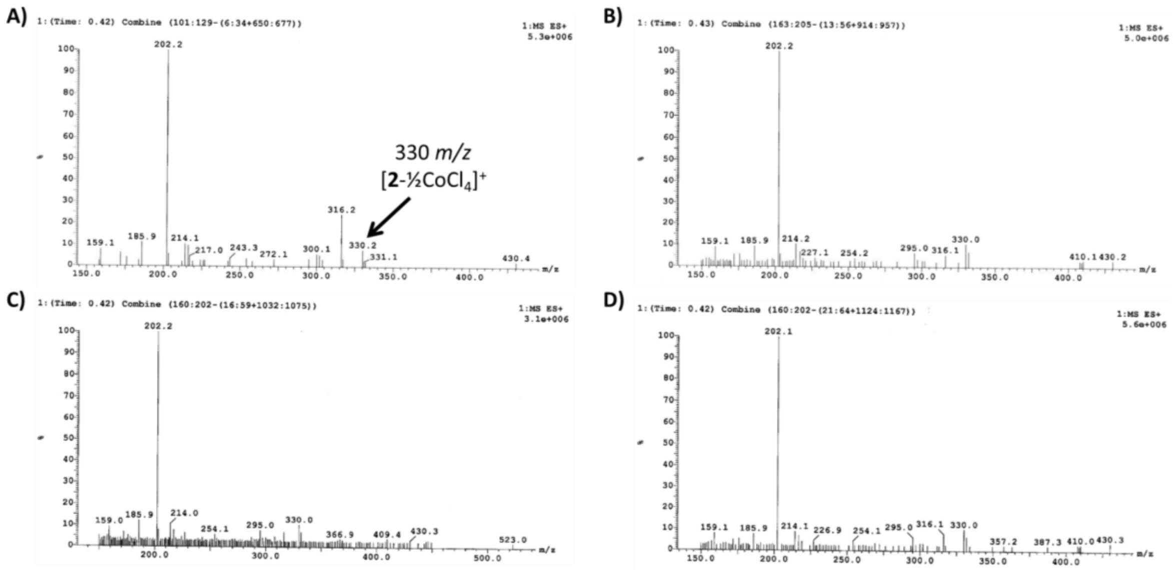

Figure S21. ESI mass spectra (positive mode) of **2** (40 μM) in H_2O:DMSO (10:1) (A) before and after incubation for (B) 24 h, (C) 48 h, and (D) 72 h at 37 ^oC.

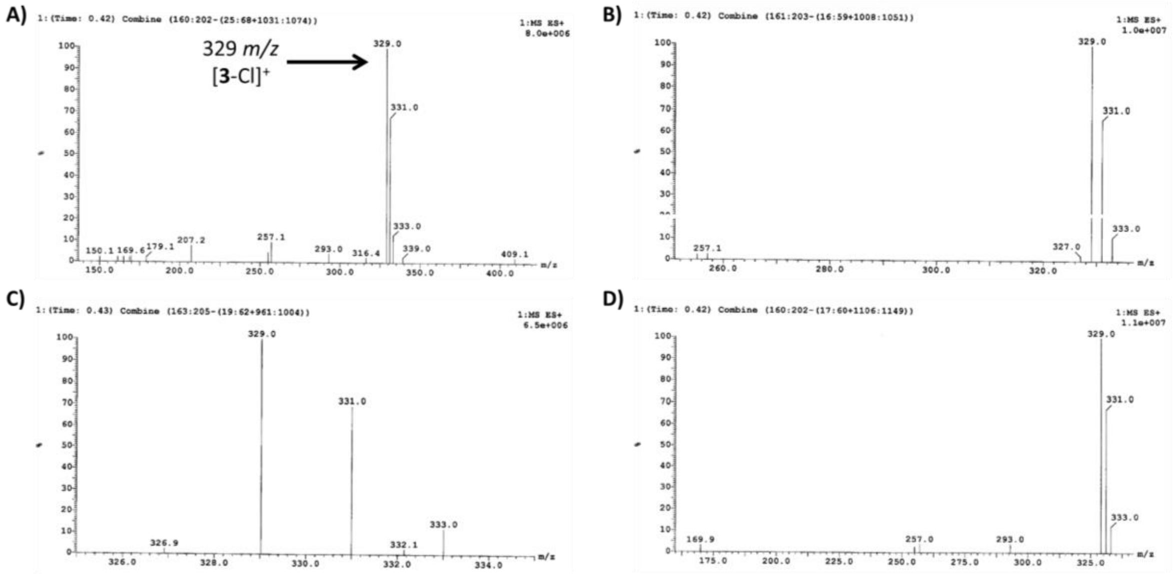

Figure S22. ESI mass spectra (positive mode) of **3** (40 μM) in H_2O:DMSO (10:1) (A) before and after incubation for (B) 24 h, (C) 48 h, and (D) 72 h at 37 ^oC.

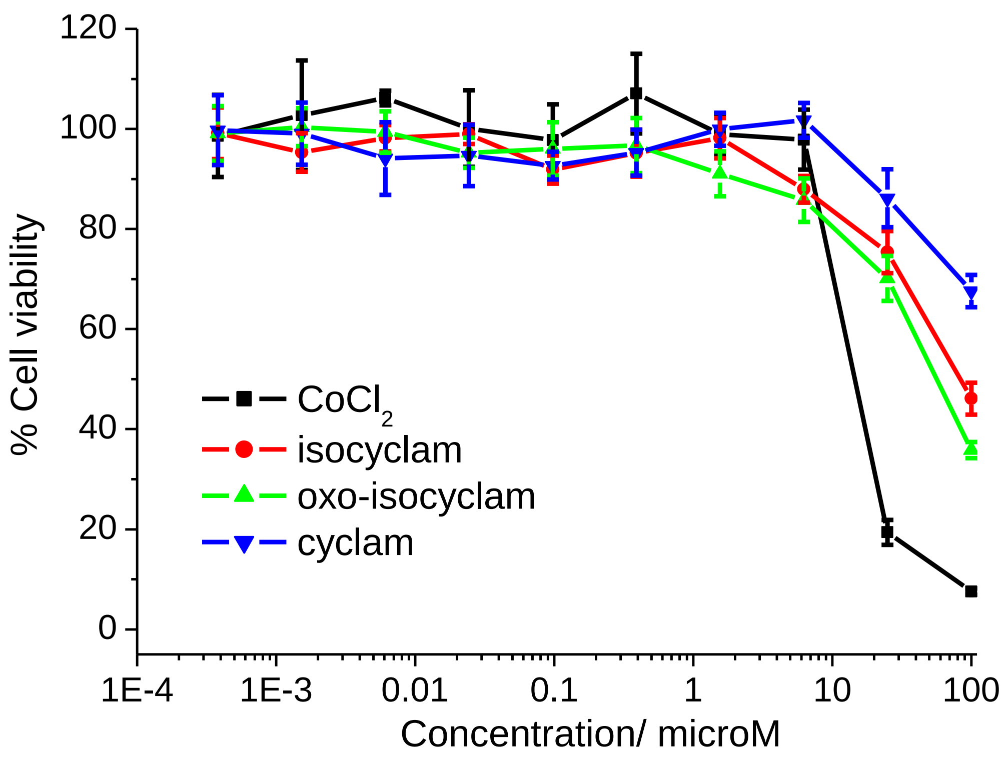

Figure S23. Representative dose-response curves for the treatment of HMLER-shEcad cells with $CoCl_2$, 1,4,7,11-tetraazacyclotetradecane, 1-oxa-4,8,12-triazacyclotetradecane, or cyclam after 72 h incubation.

Table S5. IC_{50} values of $CoCl_2$, 1,4,7,11-tetraazacyclotetradecane, 1-oxa-4,8,12-triazacyclotetradecane, and cyclam against HMLER-shEcad cells. Determined after 72 h incubation (mean of three independent experiments ± SD).

Test compound	HMLER-shEcad [μM]
$CoCl_2$	14.55 ± 0.72
1,4,7,11-tetraazacyclotetradecane	84.05 ± 12.23
1-oxa-4,8,12-triazacyclotetradecane	55.55 ± 3.46
cyclam	> 100

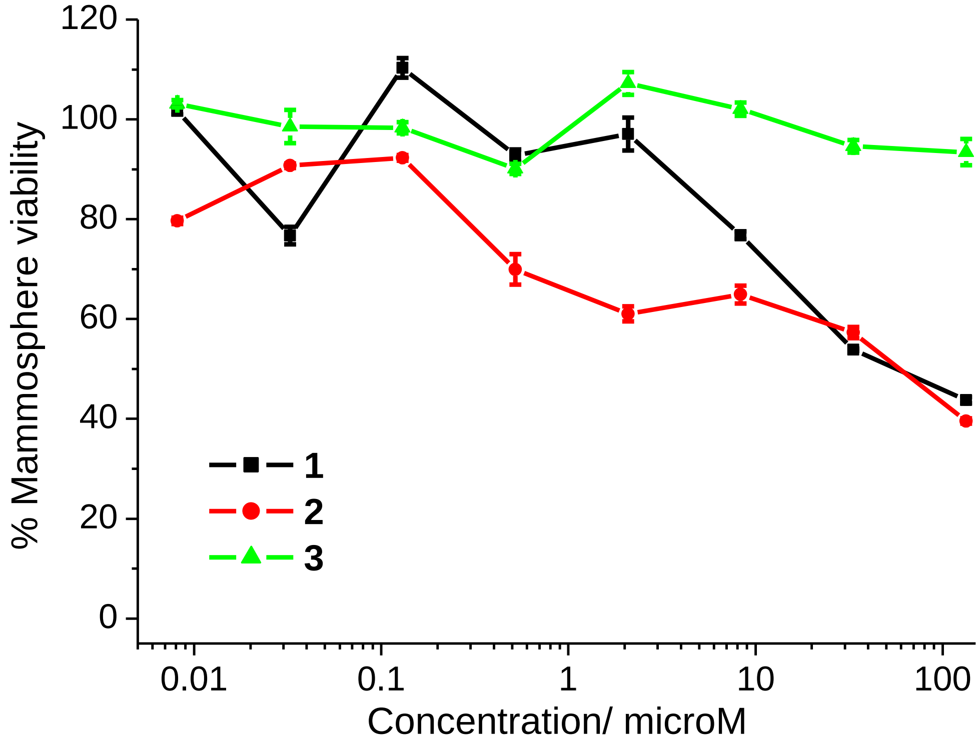

Figure S24. Representative dose-response curves for the treatment of HMLER-shEcad mammospheres with **1**-**3** after 5 days incubation.

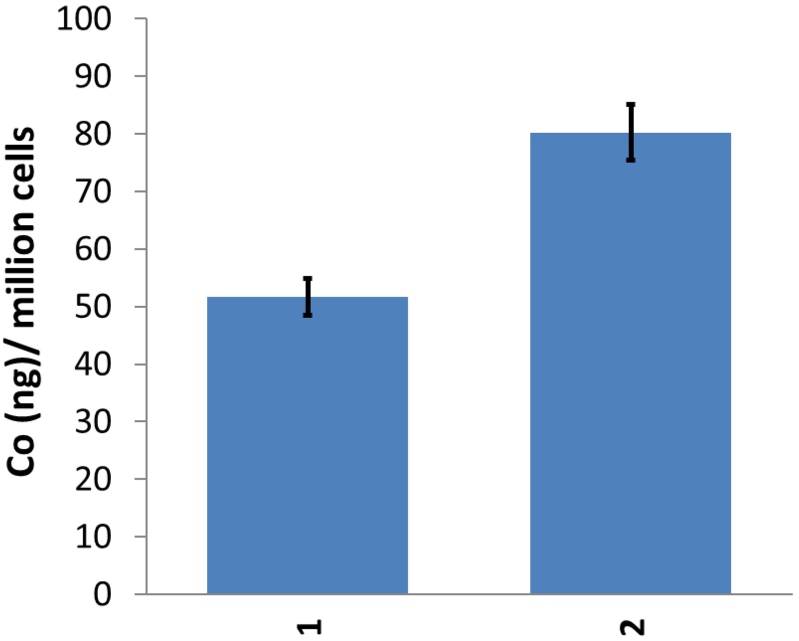

Figure S25. Cobalt content in HMLER-shEcad cells treated with **1** or **2** (2 μM for 24 h).
